# Glioma: molecular signature and crossroads with tumor microenvironment

**DOI:** 10.1007/s10555-021-09997-9

**Published:** 2021-10-23

**Authors:** Lennart Barthel, Martin Hadamitzky, Philipp Dammann, Manfred Schedlowski, Ulrich Sure, Basant Kumar Thakur, Susann Hetze

**Affiliations:** 1 Department of Neurosurgery and Spine Surgery, Center for Translational Neuro- and Behavioral Sciences, University Hospital Essen, Hufelandstraße 55, 45147 Essen, Germany; 2 Institute of Medical Psychology and Behavioral Immunobiology Center for Translational Neuro- and Behavioral Sciences, University Hospital Essen, 45147 Essen, Germany; 3 Department of Clinical Neuroscience, Osher Center for Integrative Medicine, Karolinska Institutet, Stockholm, Sweden; 4 Cancer Exosome Research Lab, Department of Pediatric Hematology and Oncology, University Hospital Essen, 45147 Essen, Germany

**Keywords:** Glioblastoma, Cancer microenvironment, Tumor microenvironment, Glioma, Cancer stem cells, Stem cells

## Abstract

In patients with glioblastoma, the average survival time with current treatments is short, mainly due to recurrences and resistance to therapy. This insufficient treatment success is, in large parts, due to the tremendous molecular heterogeneity of gliomas, which affects the overall prognosis and response to therapies and plays a vital role in gliomas’ grading. In addition, the tumor microenvironment is a major player for glioma development and resistance to therapy. Active communication between glioma cells and local or neighboring healthy cells and the immune environment promotes the cancerogenic processes and contributes to establishing glioma stem cells, which drives therapy resistance. Besides genetic alterations in the primary tumor, tumor-released factors, cytokines, proteins, extracellular vesicles, and environmental influences like hypoxia provide tumor cells the ability to evade host tumor surveillance machinery and promote disease progression. Moreover, there is increasing evidence that these players affect the molecular biological properties of gliomas and enable inter-cell communication that supports pro-cancerogenic cell properties. Identifying and characterizing these complex mechanisms are inevitably necessary to adapt therapeutic strategies and to develop novel measures. Here we provide an update about these junctions where constant traffic of biomolecules adds complexity in the management of glioblastoma.

**Graphical abstract:**

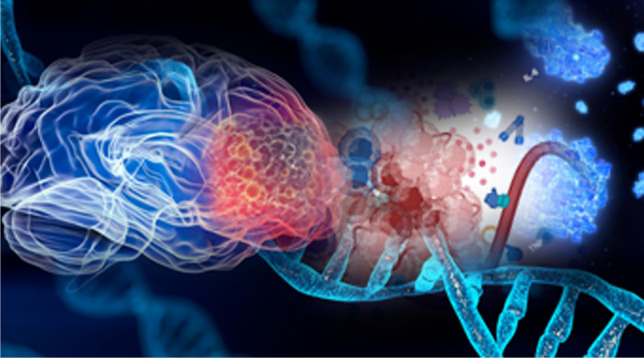

## Introduction

A curative treatment option for high-grade gliomas is currently not available. Gliomas are highly heterogeneous and proliferate invasively, making therapeutic approaches difficult. Glioma stem cells (GCSs) can develop glial tumors of varying heterogeneity with a high potential for self-renewal [1–3]. This is a critical consideration, as GCSs show greater intrinsic tolerance to therapies due to inherent characteristics and adaptive pathways of resistance than cells without stem cell properties and, thus, promote tumor recurrence [4]. As GCSs are located in all glioma areas, molecular differences can occur within a single tumor. The interaction between tumor microenvironment (TME) and tumor (stem) cells significantly influences the highly invasive tumor growth and molecular heterogeneity. Many different actors such as microglial cells, macrophages, neuronal precursor cells, vascular cells, fibroblasts, and immune cells such as dendritic cells, leukocytes, or natural killer cells take part in the TME, *inter alia* for steering of tumor characteristics like growth and diffusion but also therapy resistance [5–7]. These non-malignant cells of the circular and the lymphatic system influence the tumor neogenesis through diverse interactions such as immunological cytokine receptors exploited by the tumor cell to evade immune surveillance in TME. This “hijacking” of pro-tumor-immune cells is mediated immunologically by interleukins (IL) such as IL-6 [8], platelet-derived growth factors [9], notch-signaling pathways [10], vascular endothelial growth factor (VEGF) [11], or epidermal growth factor (EGFR) [12]. Recently, extracellular vesicles (EVs) released by the tumor cells have been shown to support the tumor *modus operandi*, to conquer the TME; additionally, cancer stem cell-derived EVs aid the reshaping of TME and influence non-stem cells in a variety of ways [13].

In addition to tumor intrinsic and extrinsic factors, hypoxia plays a crucial role in the brain tumor microenvironment. Upregulation of hypoxia-inducible factors (HIFs) has been shown to result in an aggressive and resistant phenotype of high-grade gliomas [14]. Mitochondria are the key cellular organelle to support the low oxygen availability, by switching on the oxidative phosphorylation channels. Therefore, inhibiting mitochondrial oxidative phosphorylation channels from blocking the cycle of oxygen supply in TME could be an attractive therapeutic strategy to impede tumor invasion and improve radiation response [15]. In the past 10 years, the knowledge about TME has increased significantly. However, details about the functions of the TME are not entirely understood. This also explains why the routine WHO Glioma Classification 2016 [16] will have to be further revised, as it is based on histomorphology and molecular properties only. To grade glioma for potential therapeutic decisions is of clinical relevance since differences in the composition of glioma (microenvironment) do exist between patients despite the same tumor grade, suggesting that different individual therapeutic strategies may be achieved for patients. Analyzing these complex interactions between the TME and the gliomas’ molecular properties is essential for the prognosis of individual patients.

**Fig. 1:**
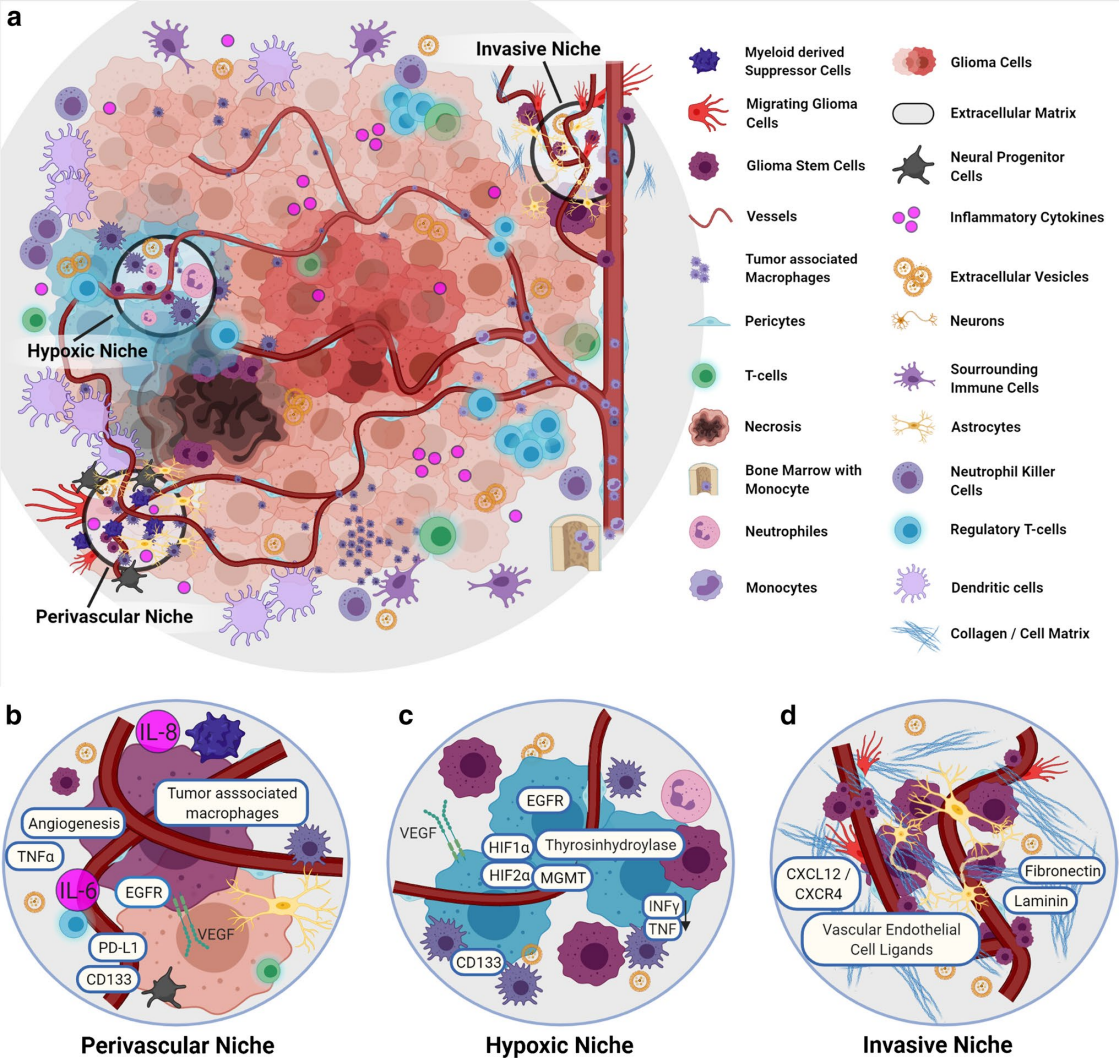
The niches of GBM niches and molecular landscape. **a** Overview of the TME of glioblastomas, with immunological players, and the three main niches that show a certain presence of specific molecular profiles. **b** The vascular niche: This niche is characterized by pronounced angiogenesis with a correspondingly increased VEGF. Here tumor macrophages are accumulated. Cytokines such as IL-6 and IL-8 are increased. Likewise, PTEN leads to increased matrix cross-linking proteins, resulting in accelerated angiogenesis. **c** The hypoxic niche contributes to glioma growth and resistance. PTEN is increased, and HIF contributes to the upregulation of VEGF and IL-8 and supports stem cell presence indicated via increased CD133. Via tyrosine hydroxylase activity, inflammatory cytokines are reduced. **d** The invasive niche: This nice is marked by a normal vessel distribution and the transition to normal brain tissue. Stem cells are associated with the vessel structure, glioma cells and microglia go along in tumor growth, and glioma stem cells are associated with endothelial cells via CXCL12/CXCR4. The cellular matrix also supports invasive tumor growth (details in text). Abbreviations: CD133, CD133–prominin 1, PROM1, is a transmembrane protein; CXCL12. C-X-C motif chemokine ligand 12; CXCR4, C-X-C chemokine receptor type 4; EGFR, epidermal growth factor receptor; HIF-1α, hypoxia-inducible factor 1-alpha; HIF-2α, hypoxia-inducible factor 2-alpha; IL-6, interleukin (6); INFy, interferon gamma; MGMT, O^6^-methylguanine–DNA methyltransferase; PD-L1, programmed death-ligand 1; PTEN, phosphatase and tensin homolog; TNF-α, tumor necrosis factor-alpha; VEGF, vascular endothelial growth factor

The molecular heterogeneity of gliomas and additional extracellular factors released by glioma cells proves to be a major hurdle for therapeutic approaches [17, 18]. Neural stem cells, oligodendrocytes, astrocytes, and immune cells are significant heterogeneous cell populations considered to interact with TME and favor tumor survival under therapy by altering invasiveness, plasticity, gene expression profile, and response towards growth factors [19], as distinct areas of the TME exhibit varying morphologically and metabolic characteristics (Fig. 1) impact tumor heterogeneity and survival [20, 21]. The tumor stem cells seem to take advantage of this altered environment, which promotes them to invade normal areas of the glioma physiologically [22].

## Molecular signatures and interactions with TME

During tumor growth, cell-intrinsic properties are in constant exchange with the surrounding tumor microenvironment. This interaction takes place not only through direct cell–cell communication but also through transmitters, chemokines, cytokines, or extracellular vesicles. This not only promotes tumor progression and invasion, as well as immune evasion, but also resistance to therapy. These interactions are summarized in relation to specific interaction mechanisms (Table 1).

**Table 1:** Summary of molecular factors significantly affecting TME and thus affecting GBM tumor growth

Molecular factor	Interaction	References
EGFR	Promotes glioma cell migration, reduces inflammatory response; induces macrophage infiltration; support neo-angiogenesis; increased in a hypoxic environment	[12, 23–25]
EGFRvIII	Supports glioma cell survival, invasion, and stemness; inflammatory triggering properties; increased sensitivity to temozolomide; macrophage infiltration; support neo-angiogenesis	[24, 26, 27]
IDH	Promotes tumor-infiltrating lymphocytes, less antitumor T-cell response; higher expression of PD-L1	[28]
IDH1mut	Favorable response to chemotherapy and radiation; reduced IFN-γ and CD8; less antitumor T-cell response	[29]
ATRX	Mutation: stabilization of the glioma cell; deletion: promotes expression of (type I) interferon	[30, 31]
KIAA1549-BRAF fusion	BRAF activation promotes pro-cancerogenic senescence via a p16 (INK4a) pathway, pro-cancerogenic TME via the CCL2/CCR2 axis; microglia recruitment	[32, 33]
NF1	NF1 incompetence: decreased cancer cell homogeneity; enhanced NF1 expression: diminished microglia activity; NF1 deactivation: increased macrophage activation	[34]
PTEN	PTEN mutation: immunosuppressive TME; PDL-1 enhancement; increased T-cell apoptosis in the presence of PTEN-deficient glioblastoma cells; absence of PTEN: immune resistance; PTEN deficiency: promoting cross-linking of proteins; supports VEGF	[35–40]
MGMT	Hypermethylation: better therapy response, promoted by hypoxic TME; reduced in presence of decreased Wnt-signaling; methylations seem to influences immune response	[41–44]
p53	Dysfunction: cell invasion and migration of glioma cells and supports inflammatory processes; loss: pro-cancerogenic activities of SASP, resulting in immunosuppressive TME; activation: immune invigoration	[45–48]
CDK4/6	Dysfunction: promotes phosphorylation of RB1, resulting in glioma cells’ division; lack of CDK4; prevents glioma cell development	[49]
RB1	Mutation: increased glioma cell proliferation rate	[50, 51]
HIF	Upregulation of VEGF and IL-8; support stem cell presence; reduction of IFN-y and TNF	[23, 52, 53]

Abbreviations: *EGFR*, epidermal growth factor receptor (vIII, variant III); *IDH1*, isocitrate dehydrogenase-(1) (mut, mutation; wt, wild type); *ATRX*, transcriptional regulator ATRX also known as ATP-dependent helicase ATRX (-mut, mutation); *KIAA1549-BRAAF*, KIAA1549 (protein-coding gene); *NF1*, neurofibromatosis type 1; *PTEN*, phosphatase and tensin homolog; *MGMT*, O^6^-methylguanine–DNA methyltransferase; *p53*, tumor protein P53 or tumor suppressor p53; *CDK4/6*, cyclin-dependent kinase 4 and 6; *RB1*, RB transcriptional corepressor 1; *HIF*, hypoxia-inducible factor

### *EGFR can support alkylating agents but also induces cell migration and an immunosuppressive TME *via* the NF-κB pathway*

**Fig. 2:**
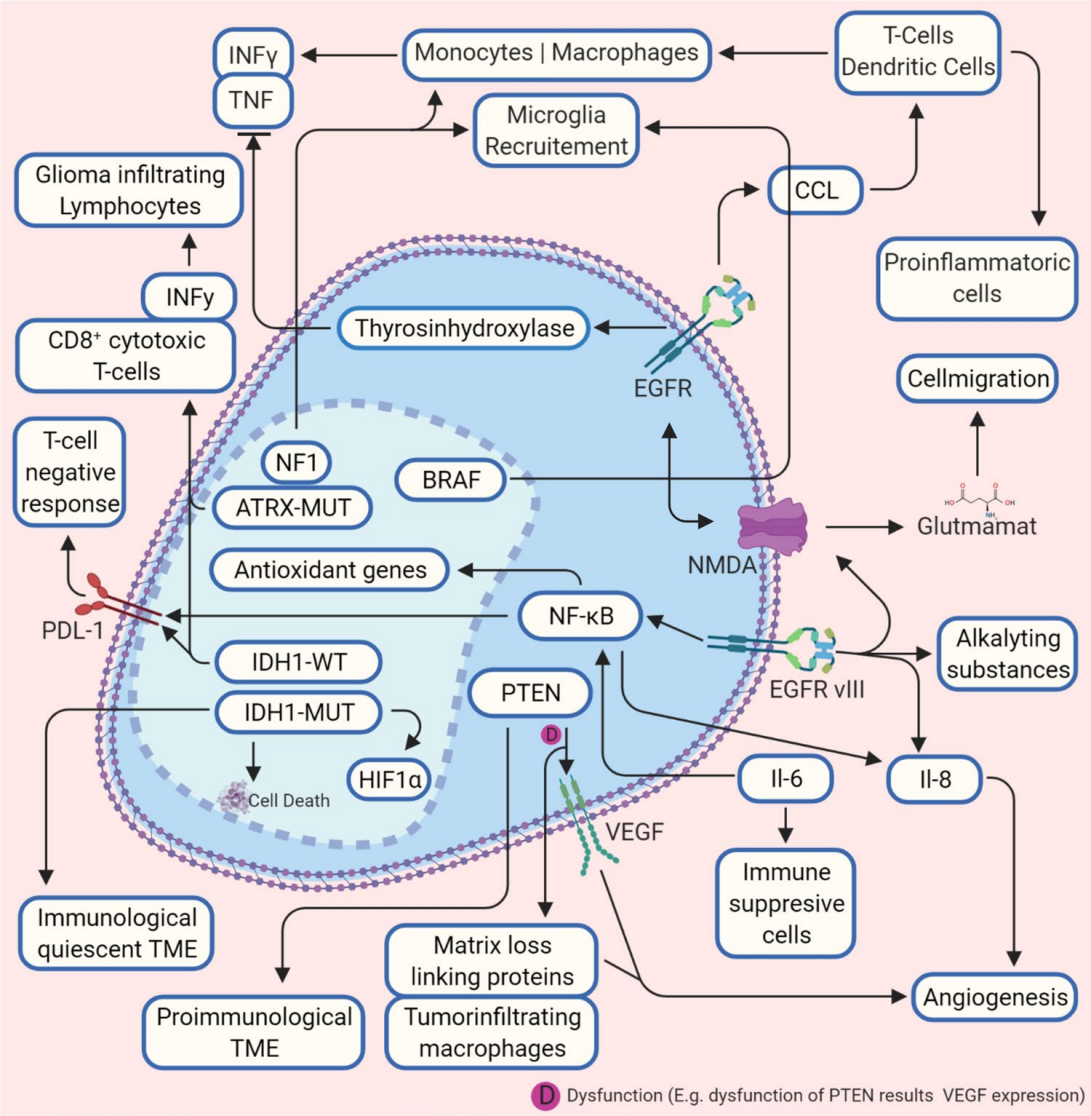
Crossroads between molecular patterns and tumor microenvironment. A diverse mechanism influences the interactions between tumor microenvironment and heterogenous molecular parameters of glioma. In this figure, the basic interactions are shown. A pro-carcinogen tumor microenvironment is promoted by an impairment of NF-gene that leads to microglia recruitment, but also BRAF promotes recruiting via CCL2. EGFR also causes a pro-cancerogenic tumor microenvironment via CCL2 by activating monocytes, whereas the recruitment of T-cells and dendritic cells supports a pro-inflammatory tumor microenvironment. EGFRvIII also activates NMDA receptors that support cell migration through glutamate release. Via CCl2 also BRAF induces microglia recruitment. IDH1-mut and ATRX promote IFN-y and CD8^+^ extracellular and IDH1-mut reduce intracellular PD-L1. In contrast, IDH1-wt promotes cell death. PTEN is crucial for tumor microenvironment composition. Deficiency of PTEN leads to increased matrix cross-linking proteins, which supports angiogenesis (also via VEGF activation) and glioma migration and tumor-infiltrating macrophages. Sufficient PTEN leads to a pro-immunological tumor microenvironment (details in the text). Abbreviations: ATRX, transcriptional regulator ATRX also known as ATP-dependent helicase ATRX (-mut, mutation); BRAF, proto-oncogene B-Raf; CCL(2), CC-chemokine-ligand-(2); EGFR, epidermal growth factor receptor (vIII, variant III); IDH1, isocitrate dehydrogenase-(1) (mut, mutation; wt, wild type); HIF-1α, hypoxia-inducible factor 1-alpha; IL-6, interleukin 6; IL-8, interleukin 8; INFy, interferon gamma; NF1, neurofibromatosis type 1; NF-κB, nuclear factor “kappa-light-chain-enhancer” of activated B-cells; NMDA, N-methyl-d-aspartate; PD-L1, programmed death-ligand 1; PTEN, phosphatase and tensin homolog; TME, tumor microenvironment; TNF-(α), tumor necrosis factor (alpha); VEGF, vascular endothelial growth factor

EGFR is a receptor tyrosine kinase located as a transmembrane protein. It is activated extracellular by ligand binding and intracellular via autophosphorylation of the kinase domain via downstream signaling pathways [26], thereby stimulating cell proliferation and survival. Tyrosine hydroxylase is induced by EGFR as shown in clonal pheochromocytoma cell line [54]; its overexpression leads to decreased interferon (IFN)-γ and tumor necrosis factor (TNF) levels as shown for lymphocytes [23], which could be a marker for poor prognosis [55], and activates the xc(-) system [56]. Xc(-) is an amino acid antiporter that regulates the exchange of extracellular l-cysteine and intracellular l-glutamate and regulates oxidative stress in various cell types [57, 58] (Fig. 2). An increased EGFR expression, in turn, leads to an increased glutamate concentration in the TME (Fig. 2), which promotes glioma cells migration via phosphorylation of the COOH terminal (carboxyl-terminus) of GluN2B (glutamate (NMDA) receptor subunit epsilon-2), resulting in increased glutamate-NMDAR-signaling [12]. Conversely, in the presence of the NMDAR-inhibitor MK-801 and sulfasalazine (an inhibitor of xc-), glioma cell migration was not observed [12]. In turn, the active mutation EGFRvIII that is transmitted into the TME from glioma cell EVs [59] acts pro-tumorigenic and supports glioma cell survival, angiogenesis, invasion, and stemness [24]. Notwithstanding, EGFRvIII is associated with increased overall survival in patients, probably caused by inflammatory triggering properties of EGFRvIII [26]. Moreover, it could be recently shown that GBM cell lines expressing endogenous EGFRvIII increased sensitivity to temozolomide in mice, which might be linked to increased DNA (deoxyribonucleic acid) mismatch repair proteins [27]. In contrast, however, temozolomide can presumably also exert selection pressure on the molecular properties of the glioma cells. For example, mutations of the mechanistic target of rapamycin (mTOR) or platelet-derived growth factor receptor (PDGFR) occur [60], leading to tumor recurrences through a “molecular resistance.” Cooperation between EGFR and EGFRvIII induces macrophage infiltration via elevated chemokine CCL2 (CC-chemokine-ligand-2) [24]. Kirsten rat sarcoma virus gene (KRAS) is involved, a monomeric G-protein and actor in signal transduction pathways for the differentiation and growth of cells. CCL2 is called a “tumor-derived chemotactic factor.” It recruits several classes of immune cells like monocytes, dendritic cells, memory T cells, and natural killer cells (Fig. 2), whereby pro-inflammatory mechanisms are modulated and neo-angiogenesis is increased [61]. Moreover, glioma expressing EGFRvIII secreting IL-6 is associated with high pro-angiogenic IL-8 [62, 63]. IL-6 activates the NF-κB (nuclear factor “kappa-light-chain-enhancer” of activated B-cells) pathway, which makes the cells less sensitive to attenuation of EGFR tyrosine kinase inhibitors [64] and induces the upregulation of programmed death-ligand (PD-L1) on peripheral myeloid cells, which promotes tumor growth in orthotopic mice models [65]. The expression of PD-L1 in high-grade glioma induces PD-L1 receptor expression in microglia, which then causes, immunosuppression in the sense of a negative T-cell response [66]. Additionally, via NF-κB signaling, antioxidant genes are regulated in cooperation with transcriptional coactivators in inflammation [67] (Fig. 2). Thus, v*ia* EGFR (vIII), glioma cell migration and survival are supported, as well as a change of the immunological state of TME in terms of a weakened proper immune response. Angiogenesis and stemness are promoted via interleukin mediation. EGFR directly influences a variety of aspects that mediate the glioma process and is a central mediator in glioma cell-TME interaction.

### Depending on the mutation status, IDH can support an anti-cancerogenic TME

The mutation status (-mut) of the genes encoding isocitrate dehydrogenase (IDH) 1/2 is of central importance for the differentiation of low-grade to higher-grade gliomas, as low-grade gliomas present higher IDH-mut rates than high-grade gliomas [68]. IDH1mut is associated with a favorable response to chemotherapy and radiation [69], and 70–90% of low-grade-glioma and secondary glioblastoma present mutations in IDH1 [70]. Non-mut-IDH converts isocitrate to α-ketoglutarate. α-Ketoglutarate is used to catalyze 2-hydroxyglutarate, an oncometabolite, and is common in IDH-mut [71]. The immune system of the TME appears to be different between IDH-mut and IDH-wt-gliomas. More tumor-infiltrating lymphocytes are present in the latter, and PD-L1 is expressed higher [28]. Interestingly, the reduced immune situation seems to be associated with an IDH-1 mutation since the expression of the genes for IFN-γ and CD8^+^ is reduced. Both genes are essential for an antitumor T-cell response [29]. This is astonishing since a weakened immune system results in a molecular subtype that goes hand in hand with a better prognosis. Derived from this, this would mean that IDH-1 wild-type tumors, for instance, most higher-grade gliomas, would be more accessible for immunotherapy. As mentioned, distinguishing low-grade and higher-grade gliomas 1p/19q-status (loss of heterozygosity (LOH)) and IDH-mut is crucial as a co-deletion of 1p/19q is typical for oligondendroglioma and essential for the differentiation against astrocytoma. Furthermore, the co-deletion indicates a better response to chemotherapy. Co-deletion of 1p/19q is mainly associated with IDH-mut, but IDH-mut does not always belong to a 1p/19q deletion [72]. It also appears that the 1p/19q status is related to the expression of integrins. These transmembrane receptors not only play a role in the adhesion of cells with the extracellular matrix but are also involved in processes such as the regulation of induced cell death, intracellular transport mechanisms, or migration in conjunction with growth factors [73]. For over 20 years, it is known that α6β4 integrin has significantly increased in human astrocytomas [74]. Recently, it was reported that in the presence of 1p/19q deletion and IDH1-mut, the expression of integrin beta-1 (ITGB1) was reduced compared to IDH1-mut, but a knockdown of α6β4 integrin in 1p/19q intact glioma cells (in vitro) and patient-derived glioma cells led to a decreased glioma development [75] (Fig. 2). IDH mutation is one of the most important genetic modification in glioma, as it supports early spread of glioma cells and, therefore, is an important early marker and therapeutic target. However, the effect on the immune-tumor microenvironment is also observed by inhibiting the T-cell response and the association with PDL-1.

### *ATRX mutation activates macrophages, natural killer cells, and neutrophils *via* IFN-γ recruitment*

ATRX (ATP-dependent helicase) and TERT (telomerase reverse transcriptase) mutations are usually present together and are essential for further differentiation of gliomas. The mutations are mostly linked to IDH-mut and an increased EGFR presence [76]. A mutation in the TERT promoter is associated with an increased TERT expression. Conversely, (inactivating) mutations for ATRX lead to a reduction of ATRX in glioma cell nuclei, which means that gene regulation via chromatin remodeling is no longer regulated [30], thereby promoting stabilization of the glioma cell. ATRX is entangled in the misregistration of immune signals in tumor cells [31]. For cell lines, it is showed that deletion of ATRX presumably promotes the expression of (type I) interferon. The authors also report better survival time in a xenograft and a syngeneic murine glioma model (ATRX KO cell line) and suspect an interaction between ATRX mutation and immune signaling in glioma cells [31] (Fig. 2). Thus, ATRX is a central player of glioma cell genesis. Depending on the mutation status, there is an influence on the stabilization of tumor cells and the presence of interferon (type I). As an important marker, ATRX is concluded in the WHO Classification 2016 (oligodendroglial *vs.* astrocytic) and seems to be involved in the mediation of TME immunological parameters.

### KIAA1549-BRAF fusion and decreased NF1 activity promotes microglia recruitment

Clinical therapies have been established with vemurafenib, a selective inhibitor of BRAF (v-raf murine sarcoma viral oncogene homolog B) and dabrafenib in treating malignant melanoma, as well as non-small cell bronchial and thyroid carcinoma. BRAF mutations are detectable in various tumors and are encoded on chromosome 7 (7q34). The protein is primarily responsible for controlling cell growth signals, including regulating the MAP kinase signaling pathway. Acquired mutations are primarily involved in the participation in tumor growth [77]. The common BRAF V600E mutation and the KIAA1549-BRAF fusion are present in gliomas [32]. The detection of a fusion gene in combination with an IDH-mut analysis is essential for clinical diagnostics, especially for the differentiation between pilocytic astrocytomas (WHO I) and diffuse astrocytomas (WHO II) [77]. A BRAV V600E mutation is primarily found in pleomorphic xanthoastrocytomas (approximately 60–70%) and gangliogliomas (about 20%) in diffuse, higher-grade gliomas [78, 79]. However, for pilocytic astrocytomas, it is reported that BRAF activation promotes pro-cancerogenic senescence via a p16 (INK4a) pathway and causes aberrant activation of the protein kinase pathway [32]. The KIAA1549-BRAF fusion creates a pro-cancerogenic TME via the CCL2/CCR2 axis, mediated by NF-κB and microglia recruitment [33] (Fig. 2).

NF1 (neurofibromatosis type 1) is a broad predisposition syndrome for various tumors, also for gliomas and most frequent tumor suppressor syndrome [80, 81], with an incidence of 1/3000 births [81] and a regulator of the RAS signaling pathways, essential for regulating cell differentiation and growth. A loss of neurofibromin expression promotes increased cell growth—as in tumors associated with NF1—via increased RAS-activation [80]. For glioblastoma cells, it has been shown that NF1 incompetence led to a decreased cancer cell homogeneity, enhanced NF1 expression led to diminished microglia activity and vice versa, and NF1 deactivation results in increased macrophage activation [34] (Fig. 2). KIAA1549-BRAF fusion is a central player in achieving cell survival and cell proliferation and is, as described, an important clinical marker for the delimitation of pilocytic astrocytoma. The influence on the CCL2/CCR2 axis described here is important for the interaction with the TME since microglia recruitment occurs and pro-cancerogenic processes are initiated. It seems particularly interesting to decipher the processes that TME mediates on the microglia to pursue new therapeutic approaches and the mechanisms in the glial cells themselves that lead to glioma-like properties. The understanding of the influence of NF1 on glioma properties as a whole and concerning the tumor microenvironment is still minimal. The effect of NF1 to induce decreased microglial activity appears to be an interesting starting point for further research. It can be assumed that immunological processes, mediated by TME, occur here since NF1 deactivation is associated with increased macrophage activation. Immune cell analyses in animal models and in vitro can be a corresponding starting point for researching these processes in more detail.

### Phosphatase PTEN activity leads to a pro-immunological TME, a reduction in macrophage recruitment and angiogenesis

The gene PTEN encodes the protein phosphatase and tensin homolog. Dysregulation of the gene is associated with different types of cancers [82]. A PTEN-deletion occurs in 30–40% of high-grade gliomas, but the impact on overall patient survival is discussed [83]. However, PTEN plays a vital role in preventing immunosuppressive TME and tumor cell evasion [35]. For glioma cells with PTEN mutation or deletion, an immunosuppressive TME evolved: the anti-cancerogenic immunity decreased, and resistance against T-cell lyses developed, together with a PDL-1 enhancement [35, 36]. In human glioblastoma cells, increased T-cell apoptosis could be detected in the presence of PTEN-deficient glioblastoma cells. Along with this, the absence of PTEN led via the PI3K-Akt-mTOR pathway to an immune resistance [35, 37]. For melanoma, it could be shown that PTEN-deficient cells encourage a resistance to immune infiltration by increased levels of cytokines, such as VEGFR and CCL2, resulting in an immunosuppressive tumor microenvironment [84, 85]. In glioma cells, PTEN deficiency fosters activation of yes-associated protein 1 (YAP1), which modulates the protein-lysine 6-oxidase (LOX) [38]. As a result, the conversion of molecules into activating aldehydes is induced, which are present in the extracellular matrix, promoting cross-linking of proteins such as elastin and collagen [39]. Tumor-infiltrating macrophages that secrete phosphoprotein 1 (SSP1) were increased via LOX activation, supporting glioma cell growth and angiogenesis. For glioblastoma cells that did not express PTEN, LOX was noticeably reduced and macrophage infiltration and tumor progression took place [38] (Fig. 2). Loss of the tumor progressor is of importance to therapeutic resistance. Still, the imparting of immunological characteristics of TME also seems to support glioma progress, such as the shift to a more immunosuppressive TME or immune resistance in the case of PTEN mutation and deficiency reveals. In addition to immune mediation, functional PTEN also appears to inhibit angiogenesis and the establishment of cross-link proteins and is, therefore, a possible key element of glioma cell stabilization.

## The hypoxic tumor microenvironment as the driving force for glioma progression

Tumor growth is selectively influenced by certain environmental properties of the tumor microenvironment. A hypoxic tumor microenvironment, which is typical for fast-growing gliomas, supports this selection pressure and establishes adaptive cell-intrinsic properties. HIF-1, for example, a typical player in tumor progression is involved, but MGMT (O-6-methylguanin-DNA-methyltransferase) methylation and the human tumor suppressor p53 are also affected by the hypoxic tumor microenvironment. These mechanisms leading to the critical progression of the tumorigenic process are summarized below (Table 1).

### *MGMT activity increases with hypoxic TME and is reduced *via* Wnt signaling*

**Fig. 3:**
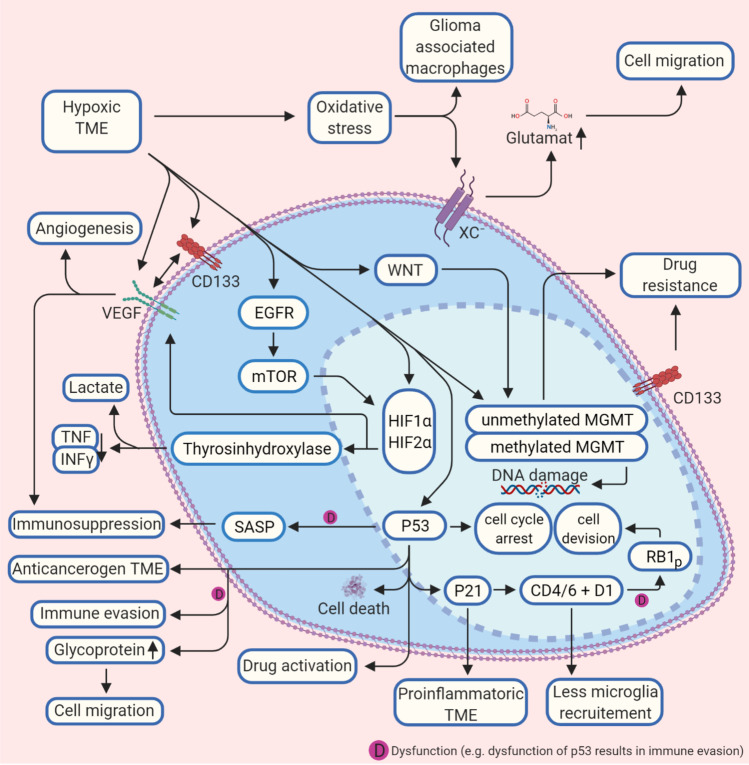
Hypoxic glioblastoma tumor microenvironment and molecular interactions. A hypoxic tumor microenvironment TME influences molecular biological processes in the glioma cell on several levels. Methylation of the MGMT, VEGF, and p53-protein are activated, which reduces the effect of alkylating agents and promotes angiogenesis. p53 is also the central regulator of p21 and CDK4/D1, which reduce microglia recruitment. A malfunction of p53 leads to increased glycoprotein concentration in the TME, which supports cell migration and immune evasion, and immunosuppression (details in the text). Abbreviations: BRAF, proto-oncogene B-Raf; CD133, CD133–prominin 1, PROM1, is a transmembrane protein; CDK4/6, cyclin-dependent kinase 4 and 6; D1, cyclin D1 protein; EGFR, epidermal growth factor receptor (vIII, variant III); HIF-1α, hypoxia-inducible factor 1-alpha; HIF-2α, hypoxia-inducible factor 2-alpha; MGMT, O^6^-methylguanine–DNA methyltransferase; INFy, interferon gamma; mTOR, mechanistic target of rapamycin; P21, cyclin-dependent kinase inhibitor 1 or CDK-interacting protein 1; P53, tumor protein P53 or tumor suppressor p53; RB1, RB transcriptional corepressor 1; SASP, senescence-associated secretory phenotype; TME, tumor microenvironment; TNF-(α), tumor necrosis factor (alpha); VEGF, vascular endothelial growth factor; WNT, Wnt signaling pathway; xc^−^, antiporter system xc^−^

DNA repair enzymes and proteins such as MGMT, ERCC (DNA excision repair protein), and APNG (alkylpurine-DNA-N-glycosylase) are also likely to be determined by extracellular mechanisms. If MGMT is hypermethylated, alkylating chemotherapy response and more prolonged survival are more likely [41]. MGMT, APNG, and ERCC1 mRNA were increased in microvesicles derived from glioma cells [86]. Understanding these mechanisms seems more important because the DNA repair enzyme MGMT inhibits the efficacy of standard clinical drugs such as temozolomide. A hypoxic TME promotes the expression of MGMT [42] (Fig. 3), and MGMT gene silencing in patients prolonged survival time under treatment temozolomide [87]. MGMT expression also depends on cellular-cellular signaling and was reduced in case of decreased Wnt signaling [43]. This downstream signal transduction pathway works in a canonical/non-canonical matter and is crucial in various diseases [88] (Fig. 3). MGMT status is crucial in the clinical evaluation of glioblastomas, as methylation status is directly associated with the response to alkylating therapies, especially in the elderly. However, a hypoxic tumor microenvironment appears to establish the therapeutically inferior MGMT status. The data on the influence of MGMT on immunological processes is still rudimentary, at least, as we have not been able to determine any valid data on this, but there are already hints that MGMT methylation and the immune response interact. Deciphering these processes is of particular clinical importance since temozolomide has immunosuppressive properties.

### Dysfunction of cell regulator p53 supports an immunosuppressed TME and immune evasion

p53, a tumor suppressor, induces the arrest of the cell cycle. Thus, a mutation of the gene that encodes the tumor suppressor protein p53 limits the prognosis in cancer [89, 90] and is present in many tumor entities and approximately 30% in glioblastomas [91]. Dysfunction of the tumor suppressor promotes cell invasion and migration of glioma cells and has a protective effect against apoptosis [92]. However, the formation of the senescence-associated secretory phenotype (SASP) is described to be p53 dependent [93] (Fig. 3), and a loss of p53 could lead to pro-cancerogenic activities of SASP [45]. Interestingly, SASP is more present in high-grade gliomas—as SASP-related genes are in these grades more expressed, and an association with IDH-wt astrocytoma is assumed [72]. SASP is involved in transforming fibroblasts into active inflammatory cells, which support tumor growth [45]. Interestingly, SASP in tumor stroma can have an immunosuppressive effect on the tumor microenvironment [46], but so far, this has only been shown for liver cells [89, 90]. On the other hand, SASP seems to support tumor progression, as it can promote epithelial-mesenchymal transition [46]. Thus, the role of SASP in glioma seems not to be finally elucidated, but it suggests that the effect of SASP is related to p53 activity [94].

Further studies show that so-called gain of function (GOF) influences mutations, which, in turn, causes inflammatory processes in the glioma TME [91] and indicates that misregulation of p53 supports inflammatory processes and also tumor-immune evasion [47, 48] (Fig. 3). Glycoproteins of the extracellular matrix (ECM) also appear to interact with p53. In TME with tumor-infiltrating leukocytes, for example, it could be shown that the pharmacological activation of p53 led to an increased anti-cancerogenic immune system and tumor regression, in addition to immune activation through the induction of immune-associated cell death [48]. For astrocytes with a p53 + / − , fibronectin and laminin are more concentrated in the ECM than in the ECM of p53 + / + astrocytes. Astrocytes with a p53 + / − were also less susceptible to apoptosis in vitro [95]. This could be due to a haploinsufficient phenotype of p53 + / − and deduce that higher-grade glioma cells create a dysfunctional TME by the reduced expression of p53 [95]. In other studies, it could be shown that the presence of laminin in the ECM in principle enables better anchoring of the cells in their environment and counteracts cell migration and invasion [96]. However, the interrelationships between glioma and TME are clearly more complex; previous studies showed that both laminin and fibronectin were involved in tumor cell migration in vitro [97]. There is currently no detailed knowledge of the interrelations between ECM proteins and the p53 status in gliomas. With mutations of the p53 gene, many interactions with compartments of the TME seem to enter, which are pro-cancerogenic but also offer approaches for new therapies (Fig. 3). p53 is certainly one of the most important known factors in eukaryotic cells to prevent their degeneration into tumor cells. Conversely, dysregulation has correspondingly many effects on cell metabolism, apoptosis, or the stability of the genome. Interactions with the TME, as we have shown in this paragraph, are also versatile. Cell invasion and migration of glioma cells are promoted in the case of altered p53, as well immunosuppression. As in many other tumors, p53 seems to be a promising starting point for glioma therapies to enable the preservation of an anti-cancerogenic TME.

### *CDK4/6 dysfunction drives cell division *via* RB1 phosphorylation*

It has been known for many years that the malfunction of the tumor suppressor protein RB1 (RB transcriptional corepressor 1) is involved in many pro-cancerogenic processes [98], and astrocytomas expression of mutated RB1 is associated with proliferation of tumor cells and is correlated to decreased survival [50]. In non-cancer cells, RB1 is dephosphorylated by the cyclin-dependent kinase 4/6 (CDK4/6) pathway and can bind the transcription factor E2F, which prevents cell division. CDK4/6 dysfunction is common in high-grade glioma [99] and promotes phosphorylation of RB1. As a result, E2F binding fails and glioma cells’ division occurs [49]. Studies using fluorescence in situ hybridization and immunohistochemistry showed a complete loss of RB1 in about 10% of glioblastoma samples examined [50]. A mutation of the 13q14 gene encoding RB1 exists in approximately 30% of all higher-grade astrocytomas [100]. p21, an inhibitor of a cyclin-dependent kinase, supports a complex between cyclin D1 and CDK4 [51]. p53 controls transcription of p21 [101] is increased by DNA damage or cell stress (for example, through a hypoxic TME) and supports physiological cell cycle arrest in increased expression levels [102]. Cyclin D1, a regulatory subunit of CDK4/6 in turn, is increasingly expressed in glioma cells [103] (Fig. 3). Furthermore, CD1 and CDK4 are critical for both the glioma cell and the TME as the lack of CDK4 prevents glioma cell development, and the absence of CD1 alone had an opposite effect [51]. Interestingly, a combined loss of CD4 and D1 led to reduced microglial activation in the TME. In many cancer entities, the CDK4/RB1 pathway is disturbed and leads to increased cell proliferation. Preclinical therapeutic approaches with inhibitors foster cell cycle arrest of the tumor cells. However, a hypoxic tumor cell environment will affect p53 and p21 as described above. The latter, in turn, influences the proper function of CDK4/6 which, together with cyclin D1, co-determine the phosphorylation status of RB1. As far as we could determine, no direct interaction between (immune-) TME and CDK4/6 is known, but at least via p53/p21 CDK4/6 seems to be indirectly influenced by the conditions of the tumor microenvironment. Consequently, CDK4/6 inhibitors could be of particular interest in glioma patients with a disturbed p53/p21 axis.

## The TME immune-state is receptive to the molecular heterogeneity of glioma and provides a protective environment for gliomas to survive and expand

Immunotherapy is a promising approach to cure glioma and can be divided into several subtypes according to the compounds applied: checkpoint inhibitors, vaccines, cytokines, CAR-T cells (chimeric antigen receptor), and monoclonal antibodies. For glioblastoma, the implementation of immunotherapies is of particular difficulty compared to solid tumors, as they are immunologically shielded by the blood–brain barrier (which can be in part disrupted through glioma growth [104], and there is evidence that the conception of immune privilege has to be reconsidered [105]). However, in principle, the host’s innate and adapted immune system shield against pro-carcinogen immunological activities. Interestingly, glioma patients seem to reveal a systematic immunodeficiency [106], and glioma itself can induce a vast immune response [107]. The prognosis for glioma is associated with the presence of immune cells in the TME [108]; for example, regulatory T cells (Tregs) are reported to be associated with a poor prognosis in cancer [107]. Nevertheless, previous infections seem to aid glioma occurrence, as gliomas, but not normal brain tissue of the same patients, are associated with human cytomegalic virus nucleic acids and proteins [109]. Non-neoplastic immune cells play a critical role in glioma of both residual and infiltrating origin. Tumor-associated macrophages count to ~ 30% of the tumor mass in glioma together with resident microglial cells [110]. The profile of residual and non-residual cells in glioma seems to be associated with distinct molecular properties. For glioma, it is reported that M2 (anti-inflammatory macrophage phenotype) is activated via pro-cancerogenic IL-10 and TGF-beta (transforming growth factor) [111]. Exosomes from glioma cells of the hypoxic niche glioma foster M2 polarization [112]. To make the occurrence of a pro-cancerogenic immune situation even more complex, the glioma immune condition is also determined by interactions with the respective molecular biological status of the tumor. For example, an increased level of tumor-associated macrophages was detectable in the case of NF1 deficiency [113]. For lower-grade glioma, it was shown that mutated EGFR was present together with an upregulated immune response [114], indicating interactions with a prognostic value between the immune microenvironment and the molecular status. Dysfunction of tumor suppressor p53 is widely known to protect tumor cells from apoptosis, and accelerating evidence reveals that dysfunction also leads to inflammatory states and tumor-immune evasion [48]. However, the critical clinical biomarker MGMT is associated with an increased immune response [44], as analyses revealed an upregulation of immune genes in unmethylated glioma. Furthermore, there are further indications that inflammatory pathways influence the molecular properties and can represent biomarkers accordingly. In the presence of the protein tyrosine phosphatase receptors-δ (PTPRD), the interleukin-1 receptor accessory protein (IL1RAP) mediates, for example, the invasion of glioma cells by supporting the development of neuronal synapses and differentiation of neurons, in vitro [115]. However, the exact interactions between immune TME and the intrinsic properties of the cell, such as the molecular biological status, have not yet been elucidated. Identifying several mechanisms promises an entirely new interpretation of individual gliomas in patients and may lead to personalized therapies to conquer glioma’s immune evasion. This is conceivable in two directions: immunotherapy based on the individual molecular status and, conversely, to modify the molecular status through targeted immunological interventions.

## Niches of the glioma, glioblastoma stem cells, and association to molecular diversity

### Scherer’s structures and niches model

The invasive growth of glioblastomas is significantly influenced by micro-anatomical areas with unique molecular properties and is crucial for stem cell differentiation, glioma cell invasion, and immune evasion. As early as 1938, the German neuropathologist, Hans Joachim Scherer, characterized the unique growth properties of gliomas concerning the white brain matter and the meninges. However, Scherer especially described perivascular tumor progress outside the Robin-Virchow areas [116]. Three major micro-anatomical niches of glioma TME can be divided, which play an essential role in the metabolic transformation of glioma cells and stem cell differentiation: the invasive, hypoxic, and perivascular niche [52] (Fig. 1). Multiple players are involved in glioma “behavior” as peripheral blood cells such as monocytes differentiate into tumor-associated macrophages and accumulate in the glioma as part of an inflammatory reaction. Furthermore, other immunological players such as inflammatory cytokines also influence glioma growth. As the tumor progresses, the glioma TME changes to an immunosuppressive environment, making immunotherapeutic approaches more difficult.

### Glioma stem cells

In all three niches, GSCs are involved in tumor development and stabilization, as well as resistance to therapies [2]. GSC’s formation and characteristics like self-replication, specialization, proliferation, and tumor induction might depend heavily on its environment. There may be very heterogeneous GSC variants in the TME, even those with glioma cell properties. In the perivascular and invasive niche, GSCs are in close contact with the excessive vascular structures and communicate with normal and aberrant blood vessels [52]. Moreover, GSCs and tumor cells can communicate with each other, thereby stabilizing themselves and supporting processes such as immune evasion that are an obstacle to tumor cell control, whereas the niches themselves appear to be dependent on the tumor entity [52]. The GSC regulation is endogenous and exogenous. In addition to metabolic regulation and genetic determinants, factors of the niches but also parameters of the TME such as the immune system are essential [2]. There are markers for many cancer stem cells, but they are variable. For GSC, the cell surface protein CD133 could be identified and was mainly represented on GSC, which showed a high proliferation [117]. However, the expression of CD133 is from the phase of the cell cycle, so that active GSCs can also display a low surface density of CD133 [2]. Other GSC markers such as integrin CD15/SSEA-1 and CD44α6 are not always reliable indicators of the actual characteristics of GSCs [2]. GSCs play a vital role in all niches for gliomas’ invasive growth, as shown in Fig. 1.

### Vascular niche

The perivascular niche with neuronal progenitor cells is characterized by increased angiogenesis and signaling protein vascular endothelial growth factor (VEGF). VEGF is supported by a mutation of the PTEN gene, which, together with EGFR, can induce increased VEGF mRNA levels [40]. However, VEGF is crucial for the enormous vascularity in glioma. It has been shown that brain tumor stem cells with the high expression level of CD133 habit also high VEGF level [118]. GSCs are also critical for tumor differentiation, which decisively influences glioma genesis due to their high differentiation potential. Those who possess the transmembrane protein CD133 contribute to angiogenesis since VEGF is associatively increased [52, 118, 119]. VEGF contributes to the blood–brain barrier breakdown by destroying tight junction proteins [120]. As a result, the brain’s privileged immune situation is lost, and peripheral immunomodulators can enter the central nervous system and the tumor more easily. The pro-inflammatory cytokine and an anti-inflammatory myokine IL-6 are associated with tissue destruction and accompanying inflammatory processes in the sense of an acute-phase reaction induced by TNF-α [121]. The cytokine aid the invasive behavior of high-grade-gliomas via PD-L1 induction via STAT-3 [65, 122], resulting in a negative T cell response. IL-8 is also a marker of pro-cancerogenic processes, supports the invasive properties of glioblastomas, and seems to mediate glioma cells’ plasticity. IL-8 is also attributed to shield glioma cells against a hypoxic environment and protects them from therapeutic stress [123]. A misdirected, IL-6-dependent NF-κB activation is associated with increased IL-8 levels [124].

### Hypoxic niche

Metabolic changes in the niche support the entire process of tumor invasion, survival, and growth. A hypoxic constitution of TME is a vast inductor of multiple pro-carcinogen pathway activities. Stem cells, also known as tumor-initiating cells, seem to adapt remarkably well to the hypoxic, lactate-rich environment, promoting tumor growth [25]. A central component of the regulation associated with HIF 1-alpha and HIF-2 alpha in the hypoxic niche represents angiogenesis and resistance to the acidotic environment, among other things, through the upregulation of VEGF and IL-8 and also supports stem cell presence as well as CD133, which is also increased in cells in hypoxic environments [52, 53]. Crucial pathways such as WNT are also upregulated in cells in a hypoxic environment and are associated with the unmethylated MGMT type, chemotherapeutic resistance, and reduced induction of cell cycle arrest, as described above. The hypoxic environment also leads to increased EGFR activity, which induces mTOR HIF [25]. Therefore, it could be assumed that mTOR-inhibiting chemotherapeutic agents can have a good advantage, especially on glioma cells in a hypoxic environment. Additionally, immunological processes are triggered by hypoxic conditions. The HIF expression induces activation of tyrosine hydroxylase [125], and a reduction of IFN-y and TNF can occur, which has been shown for lymphocytes [23]. A reduction in IFN-y has a pro-cancerogenic effect since it promotes apoptosis, restrains cell proliferation (shown in lung carcinoma), and has an antiproliferative influence on glioblastoma growth in cell lines [126, 127]. In turn, prolyl hydroxylase domain enzymes (PHD), which α-KG and Fe2^+^ require, are essential for the degradation and hydroxylation of HIF. In IDH-mut cells, there are decreased levels of α-ketoglutaric acid and Fe2^+^, which would support HIF [68]. In hypoxic TME areas, a glycolytic metabolism is supported, resulting in increased lactate and H^+^ ion levels. Both acidosis and hypoxia can affect the essential cellular organelle mitochondria by altering glycolysis [22, 128]. Thus, investigation of the exact mechanisms of glioma cell—TME interactions under hypoxic metabolic circuit—might increase the chances of developing new effective therapies.

### Invasive niche

An essential feature of glioblastomas is their highly invasive growth. Glioblastomas do not metastasize through lymphatic systems or vascularly. The spreading occurs along the vessels’ course; here, glioma cells displace the vascular astrocytes regardless of the vessel size [129]. GSCs stimulated by ligands such as notch, angiopeptin, or endocrine are released from vascular endothelial cells [60]. This is supported by the CXC chemokine receptor type 4 (CD184), encoded by the CXCR4 and the stromal cell-derived factor 1 (CXCL12). The CXCL12/CXCR4 axis drives cell proliferation, angiogenesis, and glioma invasion. Glioma stem cells are associated with endothelial cells using the CXL12/CXCR4 axis. Mediated by TGF-ß, the transformation to pericytes then takes place. Conversely, eradicating these pericytes inhibits the glioma process along the vessels by reducing neovascularization [130]. A current study showed that the forkhead box protein M1, a transcription factor involved in physiological cell division and cell cycle regulation [131], is increasingly expressed via the CXCL12/CXCR4 axis, thereby increasing resistance to temozolomide [132].

## Extracellular vesicles: a novel emerging player in the context of glioma TME modulation

EVs are released from cells and, thus, also from cancer cells, carrying functional proteins, small RNAs (ribonucleic acid), and DNA from the donor cells [133]. They are essential for intercellular communication and take part in the regulation of physiological processes. EVs are the heading for exosomes (50–200 nm, apoptotic bodies (50–2.000 nm), microvesicles (< 100–1 μm), and oncosomes (> 1 μm), and are important for cell–cell interactions in TME and via EV communication. Cell properties like phenotype and functions can be influenced and are, thus, involved in building up a pro-cancerogenic environment and resistance to therapy [134]. Tumor suppression signaling such as miR-1, a microRNA precursor, is mediated by EVs. The expression of miR-1 leads to reduced neovascularization and glioma spread [135]. Nevertheless, EVs also stimulate non-cancerogenic astrocytes to switch to a pro-cancerogenic phenotype, achieved via p53 and Myc-gene [136]. A further example, the expression of EGFRvIII alters the expression of genes that regulate EV procession [137] and comprises both the active and not active EGFR variant [59, 138]. Moreover, CSF-derived EV revealed mutations in EGFR [139]. It is likely that through EVs, tumor niches are established, as active VEGFR was shown to be carried in tumor-shed vesicles [140]. However, determining the interactions between EVs and the TME is important for developing new therapeutic strategies and, if necessary, implementing anticancer mechanisms in the TME and glioma cell via EV transfer. As EVs carry the genetic properties of the original cell, they are a valuable diagnostic tool in the sense of a liquid biopsy.

## Importance for therapeutic approaches

The interactions between intrinsic properties of glioma cells and their microenvironment strongly influence the treatment responses. The tumor heterogeneity and immunosuppressive situation listed in this review are crucial for this concern. Although the immunosuppressive TME is important for the growth and evasion of gliomas, they show a calm immune situation compared to other tumors. Gliomas have, for example, only a few tumor-infiltrating T-cells and decreased PD-L1 expression levels [141]. The secretion of several different cytokines and chemokines from the glioma cells themselves can variably influence the immune microenvironment, for example, by altering the macrophage polarization, T-cell and natural killer cell activity, or dendritic cell maturation [142]. This causes immunotherapeutic approaches to become more difficult; however, these approaches are promising. In the last few years, many immunotherapeutic studies have been performed, comprising oncolytic virotherapies (in situ vaccination), dendritic cell vaccines or peptide vaccines (peripheral vaccination), checkpoint inhibitors, and chimeric T-cell receptors. In Table 2, representative clinical trials are listed.

**Table 2:** Representative clinical trials of immunotherapies for glioblastoma

Approach	Phase	Completed/ongoing	Sample size	PFS(m)	OS(m)	Year published	References
**Adaptive T-cells**							
**CAR-T cells (IL13Rα2)**	I	Completed	3	NR	11	2015	[143]; NCT00730613
Assessment of the feasibility and safety of cellular immunotherapy utilizing ex vivo expanded autologous CD8-positive T-cell clones genetically modified to express the IL-13 zetakine chimeric immunoreceptor and the Hy/TK selection/suicide fusion protein in patients with recurrent or refractory, high-grade malignant glioma							
**T-cells (HER2-CAR-CMV)**	I	Completed	16	3.5	24.5	2017	[144]; NCT01109095
To evaluate the safety of escalating doses of autologous CMV-specific cytotoxic T-lymphocytes (CTL) genetically modified to express chimeric antigen receptors targeting the HER2 molecule in patients with HER2-positive glioblastoma multiforme, who have recurrent or progressive disease after front line therapy							
**T-cells (CMV specific)**	I	Completed	19	8.2	13.3	2014	[145]; ACTRN12609000338268
Assessment of the safety and tolerability of autologous CMV-specific T-cell therapy for recurrent GBM							
**Immuncell-LC-T-cells**	III	Completed	180	8.1	22.5	2017	[146]; NCT00807027
Assessment of the superiority of INNOCELL Corp. “Immuncell-LC” in aspects of therapeutic efficacy and safety when administered with temozolomide to glioblastoma patients when compared with the control group who did not receive administration of the drug							
**Checkpoint inhibitors**							
**Ipilimumab (BMS-734016)**	II	Completed	72	NR	7/4	2012	[147]; NCT00623766
Assessment of the response of melanoma with brain metastases to ipilimumab treatment while maintaining acceptable tolerability							
**Nivolumab, anti-PD-1 antibody**	III	Active, not recruiting (last update posted: April 19, 2021)					NCT 02,017,717
Comparison of the efficacy and safety of nivolumab administered alone *versus* bevacizumab in patients diagnosed with recurrent, and to evaluate the safety and tolerability of nivolumab administered alone or in combination with ipilimumab in patients with different lines of GBM therapy (CheckMate143)							
**Nivolumab, anti-PD-1 antibody**	III	Active, not recruiting (last update posted: February 3, 2021)					NCT02617589
Evaluation of patients with glioblastoma that is MGMT unmethylated (the MGMT gene is not altered by a chemical change). Comparison with patients receiving standard therapy with temozolomide in addition to radiation therapy (CheckMate498)							
**Nivolumab, anti-PD-1 antibody**	III	Active, not recruiting (last update posted: September 11, 2020)					NCT02667587
Evaluation of patients with glioblastoma that is MGMT methylated (the MGMT gene is altered by a chemical change). Patients will receive temozolomide plus radiation therapy. They will be compared to patients receiving nivolumab in addition to temozolomide plus radiation therapy (CheckMate548)							
**Vaccines**							
**IMA950-vac**	I	Completed	45	NR	15.3	2016	[148]; NCT01222221
Aim of the study was to elucidate the side effects of vaccine therapy when administered together with temozolomide and radiation therapy in treating patients with newly diagnosed glioblastoma multiforme							
**DCs vaccine**	II	Completed	26	12.7	23.4	2017	[149]; NCT01006044
Investigation of efficacy and safety of autologous dendritic cell vaccination in glioblastoma multiforme patients after complete surgical resection with a fluorescence microscope							
**CDX-110 (rindopepimut)**	III	Completed	745	8	20.1	2017	[150]; NCT01480479
Investigation whether adding of the experimental vaccine rindopepimut (also known as CDX-110) to the commonly used chemotherapy drug temozolomide can help improve the life expectancy of patients with newly diagnosed, resected EGFRvIII positive glioblastoma. CDX-110 was admixed with granulocyte macrophage-colony stimulating factor							
**CDX-110 (rindopepimut)**	II	Completed	85	5.5	21.8	2015	[151]; NCT00458601
Evaluation of CDX-110 vaccination when given with standard of care treatment (maintenance temozolomide therapy). Study treatment was given until disease progression. Follow-up for long-term survival information. Efficacy was measured by the progression-free survival status at 5.5 months from the date of the first dose. CDX-110 was admixed with Granulocyte macrophage-colony stimulating factor							
**Dendritic cell (DC)-based vaccine (targeting cancer stem cells)**	I	Completed	20	23.1	25.5	2013	[152]; NCT00846456
Evaluation of immunological response, time to disease progression and survival time (time frame: five years)							
**GP96 heat shock protein-peptide complex**	I/II	Completed	41	4.5	9.5	2014	[153]; NCT00293423
Investigation of the side effects and best dose of gp96 heat shock protein-peptide complex vaccine to see how well it works in treating patients with recurrent or progressive high-grade glioma over time							
**Survivin peptide mimic SurVaxM (SVN53-67/M57-KLH)**	I	Completed	9	17.6	86.6	2016	[154]; NCT01250470
Studying the side effects of vaccine therapy when given together with sargramostim in treating patients with malignant glioma							
**Cytomegalovirus pp65-targeted vaccination**	I/II	Completed	11	25.3	41.1	2017	[155]; NCT00639639
Studying how well vaccine therapy works in treating patients with newly diagnosed glioblastoma multiforme recovering from lymphopenia caused by temozolomide							
**GVAX vaccine**	I	Completed	11	NR	8.8	2016	[156]; NCT00694330
Aim was to test the safety of vaccination of cells called GM-K562 cells mixed with the participant’s own irradiated tumor cells							
**DCVax®-L**	III	First results published	331		34.7/19.8	2018	[157]; NCT00045968; NCT02146066
Investigation of the efficacy of an investigational therapy called DCVax(R)-L in patients with newly diagnosed GBM for whom surgery is indicated (NCT00045968) Open-label expanded access to study for patients for whom the vaccine was manufactured during the Northwest Biotherapeutics’ 020,221 DCVax-L for GBM screening process, but they subsequently failed to meet specific enrollment criteria (NCT02146066)							
**NOA-16**	I	Completed	39			2021	[158]; NCT02454634
Evaluation of safety and tolerability of and immune response to the IDH1 peptide vaccine in patients with IDH1R132H-mutated, WHO grade III–IV gliomas							
**DNX-2401 (formerly known as delta-24-RGD-4C)**	I/I	Completed/recruiting (last update posted: September 28, 2021)	37/		9.5/	2018/	[159]; NCT00805376; NCT03895658
Aim was to find the highest tolerable dose of DNX-2401 that can be injected directly into brain tumors and into the surrounding brain tissue where tumor cells can multiply. A second goal was to study how the new drug DNX-2401 affects brain tumor cells and the body in general							
**Personalized neoantigen cancer-vaccine-wRT**	I/Ib	Recruiting (last update posted: May 21, 2021)					NCT02287428
Studying a new type of vaccine as a possible treatment for patients with glioblastoma. Tests the safety of an investigational intervention and tries to define the appropriate dose of the intervention to use for further studies							
**PVSRIPO**	I/II	Active, not recruiting (last update posted: May 21, 2021); data are published	61			12.5	NCT01491893
The aim is to determine the maximally tolerated dose (MTD) and the recommended phase 2 dose (RP2D) of PVSRIPO when delivered intracerebrally by convection-enhanced delivery (CED)							

Abbreviations: *ACTRN*, Australian clinical trials registration number; *CMV*, cytomegalovirus; *CMV pp65*, cytomegalovirus phosphoprotein 65 RNA; *DCs*, dendritic cells; *EGFR*: epidermal growth factor receptor (vIII: variant III); *GBM*: glioblastoma multiforme; *GVAX*, cancer vaccine composed of whole tumor cells; *HSPPC-96*, heat shock protein-peptide complexes 96; *IDH*, isocitrate dehydrogenase; *MGMT*, O^6^-methylguanine–DNA methyltransferase; *NCT*, ClinicalTrials.gov Identifier; *OSm*, overall survival (m, months); *PFSm*, progression-free survival (m, months); *PVSRIPO*, modified poliovirus. The status of the studies was last checked on September 28, 2021 (https://clinicaltrials.gov/)

The current guideline to treat glioblastomas is still primarily dependent on histopathological, molecular biological, and clinical/radiological evaluation. The EANO (European Association of Neuro-Oncology) issued new recommendations based on novel studies at the end of 2020 [160]. It is also recommended that steroids, which are widely used in clinical practice for the treatment of edema, should not be given if the patient is asymptomatic or has minimal symptoms. If the administration is required, the medication should be discontinued as soon as possible since immunosuppressive steroids can worsen the immunological properties of the gliomas and may impact the efficacy of radiotherapy, chemotherapy, and immunotherapy. Glioblastomas have so far only responded to a limited extent to immunotherapy. Therefore, according to EANO, immunotherapies are currently not recommended in clinical routine (Table 3). The standard therapy is still temozolomide and/or radiation therapy. Bevacizumab, a humanized monoclonal antibody (subtype IGg1) directed against VEGF, can be used in the case of progression or recurrence of glioma [160].

**Table 3:** Current therapy recommendations according to the EANO (European Association of Neuro-Oncology; 2020) for adult patients with common diffuse gliomas (table adapted to [160])

Histopathological/molecular	Initial treatment at the time point of diagnosis	Treatment in case of progression or recurrence
	Total gross resection is recommended whenever a safe operation is possible in all patients with newly diagnosed gliomas	Depending on tumor-board recommendation, a second surgery should be considered. The indication for a second radiation is controversial
Glioblastoma, NOS WHO grade 4	Temozolomide and radiotherapy (54–60 Gy in 1.8–2-Gy fractions) Age > 65–70 years and MGMT unmethylated glioblastoma: radiotherapy (40 Gy in 2.67-Gy fractions) Age > 65–70 years and MGMT methylated glioblastoma: temozolomide and radiotherapy or temozolomide	Nitrosourea and temozolomide. Possible approach with bevacizumab (depending on local availability) A radiotherapy can be initiated for patients that have been not previously treated with radiotherapy
Glioblastoma, IDH wild type, WHO grade 4; giant cell glioblastoma; gliosarcoma; epithelioid glioblastoma	Same recommendation as for NOS glioblastomas Tumor-treating fields remain controversial when they are used in a temozolomide maintenance setting (despite positive results in a phase III study)	Same recommendation as for NOS glioblastomas
Diffuse midline glioma, H3 K27M mutant, WHO grade 4	Radiotherapy (54–60 Gy in 1.8–2-Gy fractions) and chemotherapy with temozolomide	Nitrosourea and temozolomide. Possible approach with bevacizumab (depending on local availability)
Diffuse hemispheric glioma, H3.3 G34 mutant, WHO grade 4	Temozolomide and radiotherapy	Same recommendation as for diffuse midline glioma

Abbreviations: *Gy*, gray; *IDH*, isocitrate dehydrogenase; *MGMT*, O6-methylguanine–DNA methyltransferase; *NOS*, not otherwise specified; *WHO*, World Health Organization

However, the initiation of adaptive immune responses for the treatment of glioblastoma seems promising, as shown in the clinical studies (Table 2). In some cases, longer overall survival rates could be associated with the therapies. Nevertheless, the immunosuppressive TME, in combination with the heterogeneity of the gliomas, seems to have prevented a decisive immunotherapeutic breakthrough so far. To establish new, effective therapeutic approaches, it is even more important to understand the interplay of intrinsic (molecular) properties and the associated immune profiles of TME.

## Conclusions and future directions

**Fig. 4:**
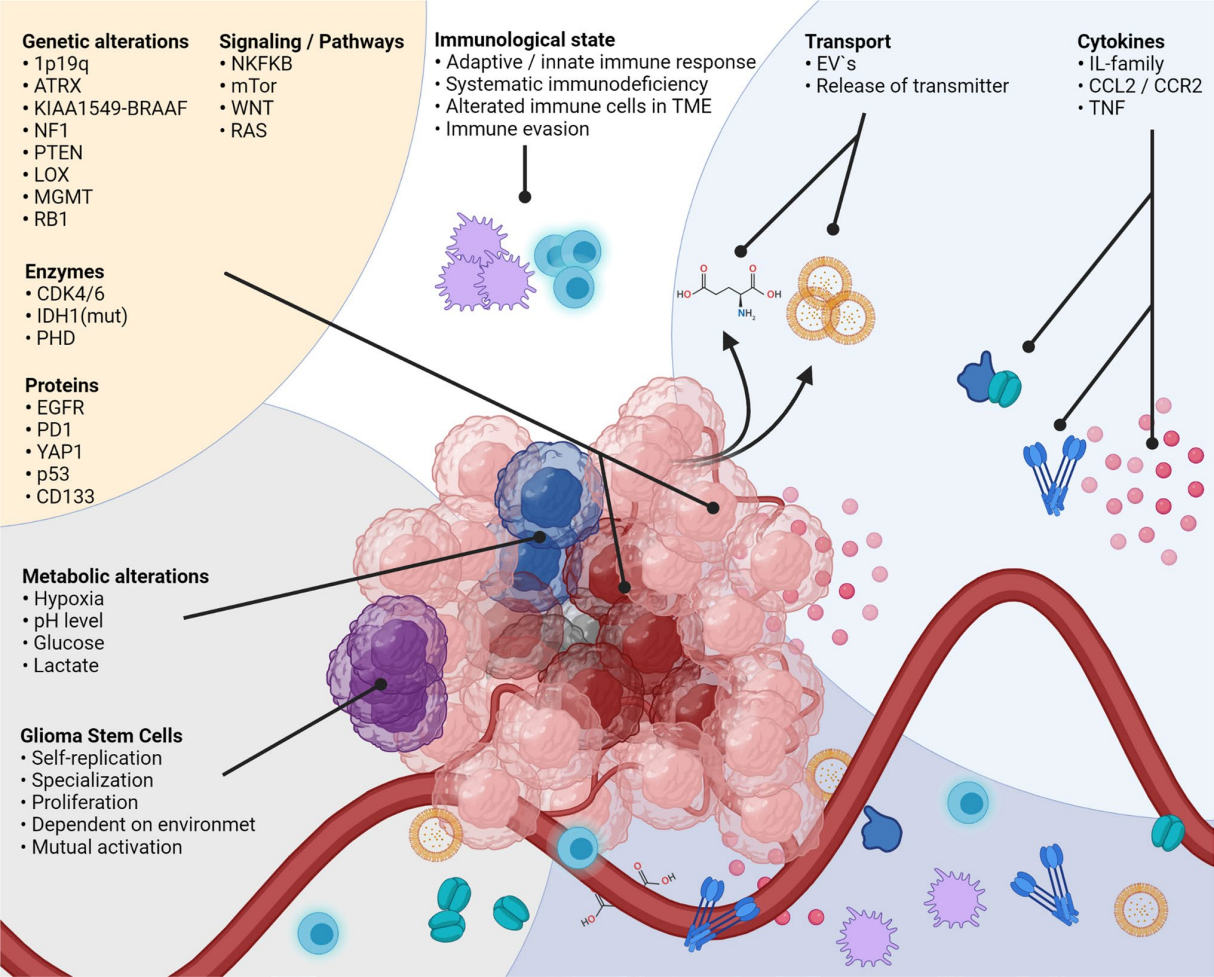
The interactions between TME and glioma cells are complex, as the multiple players of widespread origin show. Intracellular factors, pathways, cytokines, genetic alterations, or environmental properties are involved, and the molecular characteristics of glioma cells are dependent on these parameters. Furthermore, vice versa, the glioma molecular patterns influence the TME composition. The detailed interactions are listed in the text. Abbreviations: 1p19q, combined loss of the short-arm chromosome 1 (i.e., 1p) and the long-arm chromosome 19 (i.e., 19q); ATRX, transcriptional regulator ATRX also known as ATP-dependent helicase ATRX (-mut, mutation); BRAF (human gene that encodes a protein called B-Raf); CCL2, CC-chemokine-ligand-2; CCR2, C–C chemokine receptor type 2; CDK4/6, cyclin-dependent kinase 4 and 6; CD133, CD133–prominin 1, PROM1, is a transmembrane protein; EGFR, epidermal growth factor receptor (vIII, variant III); EVs, extracellular vesicles; IDH1, isocitrate dehydrogenase-(1) (mut, mutation; wt, wild type); IL-family, interleukin family; KIAA1549-BRAAF, KIAA1549 (protein-coding gene); LOX, lysyl oxidase, also known as protein-lysine 6-oxidase; MGMT, O^6^-methylguanine–DNA methyltransferase; mTOR, mechanistic target of rapamycin; NF1, neurofibromatosis type 1; NF-κB, nuclear factor “kappa-light-chain-enhancer” of activated B-cells; P53, tumor protein P53 or tumor suppressor p53; PD-L1, programmed death-ligand 1; PHD, prolyl hydroxylase domain enzymes; PTEN, phosphatase and tensin homolog; RAS, RAS proteins control signaling pathways that are key regulators of normal cell growth and malignant transformation; RB1, RB transcriptional corepressor 1; TME, tumor microenvironment; TNF, tumor necrosis factor; WNT, Wnt signaling pathway; antiporter system xc^−^

The TME plays a crucial role in glioma initiation, formation, and differentiation, and influences molecular characteristics, as the number of publications and the substantial progress show. Understanding communication processes between TME, GCSs, and developed glioma cells is indispensable to identify new therapeutic targets and treatment regimens against glioma and rethink old therapy strategies. Moreover, it seems evident that genetic sub-variants of gliomas probably require different therapeutic approaches, as pro-cancerogenic processes are promoted via cell-TME-cell interactions. The players involved in TME-glioma are of multiple origins, and, in many cases, it is not possible to assign properties to them that inhibit or promote glioma growth (Fig. 4). At present, the first-line therapy with temozolomide of glioblastoma does not vary between patients, and the vast molecular heterogeneity and prognostic factors, even within a single glioma, are not considered. To offer personalized therapies, it seems inevitable for each patient to decipher the individual molecular peculiarities. The molecular landscape of glioma may not be adequately described by a neuropathological analysis in clinical routine alone, as limited areas of the glioma are examined in tumor samples collected during surgery. However, since molecular differences occur within the glioma, the investigation of circulating tumor DNA and exosomes is a promising starting point for molecular screening, in conjunction with cytokine analyses, to reveal immunological pathways that are influencing disease progression. In addition to therapy optimization, a molecular and inflammatory glioma profile that has been compiled comprehensively in this way can also help to decipher why individual gliomas recur and others do not. Moreover, the use of engineered EVs to activate anti-cancerogenic cell properties is a promising approach to target direct mechanisms that promote glioma growth in distinct glioma heterogeneities, extra- and intracellular. However, knowledge of these dependencies is still very limited. Therefore, current glioma TME research should focus on the composition of TME during various glioma stages and glioma subspecies.

## Data Availability

Not applicable.

## References

[1] Hadjipanayis CG, Van Meir EG (2009). Tumor initiating cells in malignant gliomas: Biology and implications for therapy. Journal of Molecular Medicine.

[2] Lathia JD, Mack SC, Mulkearns-hubert EE, Valentim CLL, Rich JN (2015). Cancer stem cells in glioblastoma. Genes and Development.

[3] Prager BC, Bhargava S, Mahadev V, Hubert CG, Rich JN (2020). Glioblastoma stem cells: Driving resilience through chaos. Trends in Cancer.

[4] Osuka S, Van Meir EG (2017). Overcoming therapeutic resistance in glioblastoma: The way forward. The Journal of Clinical Investigation.

[5] Cole AP, Hoffmeyer E, Chetty SL, Cruz-Cruz J, C.-, Hamrick, F. H., Youssef, O., Cheshier, S., & Mitra, S. S.  (2020). Microglia in the brain tumor microenvironment. Advances in experimental medicine and biology.

[6] Goenka, A., Tiek, D., Song, X., Huang, T., Hu, B., & Cheng, S. Y. (2021). The many facets of therapy resistance and tumor recurrence in glioblastoma. *Cells*, *10*(3). 10.3390/cells10030484 10.3390/cells10030484 PMC7995978 33668200

[7] Vitale I, Manic G, Coussens LM, Kroemer G, Galluzzi L (2019). Macrophages and metabolism in the tumor microenvironment. Cell Metabolism.

[8] Cheng W, Ren X, Zhang C, Cai J, Liu Y, Han S, Wu A (2016). Bioinformatic profiling identifies an immune-related risk signature for glioblastoma. Neurology.

[9] Ko EA, Lee H, Sanders KM, Koh SD, Zhou T (2020). Expression of alpha-type platelet-derived growth factor receptor–influenced genes predicts clinical outcome in glioma. Translational Oncology.

[10] Bazzoni R, Bentivegna A (2019). Role of notch signaling pathway in glioblastoma pathogenesis. Cancers.

[11] Osterberg N, Ferrara N, Vacher J, Gaedicke S, Niedermann G, Weyerbrock A, Doostkam S, Schaefer H-E, Plate KH, Machein MR (2016). Decrease of VEGF-A in myeloid cells attenuates glioma progression and prolongs survival in an experimental glioma model. Neuro-Oncology.

[12] Suina K, Tsuchihashi K, Yamasaki J, Kamenori S, Shintani S, Hirata Y, Okazaki S, Sampetrean O, Baba E, Akashi K, Mitsuishi Y, Takahashi F, Takahashi K, Saya H, Nagano O (2018). Epidermal growth factor receptor promotes glioma progression by regulating xCT and GluN2B-containing N-methyl-d-aspartate–sensitive glutamate receptor signaling. Cancer Science.

[13] Su C, Zhang J, Yarden Y, Fu L (2021). The key roles of cancer stem cell-derived extracellular vesicles. Signal Transduction and Targeted Therapy.

[14] Fuchs, Q., Pierrevelcin, M., Messe, M., Lhermitte, B., Blandin, A.-F., Papin, C., Coca, A., Dontenwill, M., & Entz-Werlé, N. (2020). Hypoxia inducible factors’ signaling in pediatric high-grade gliomas: Role, modelization and innovative targeted approaches. *Cancers*, *12*(4). 10.3390/CANCERS12040979 10.3390/cancers12040979 PMC7226233 32326644

[15] Molenaar, R. J. (2011). Ion channels in glioblastoma. *ISRN Neurology*, 1–7.10.5402/2011/590249 10.5402/2011/590249 PMC3263536 22389824

[16] Louis DN, Perry A, Reifenberger G, von Deimling A, Figarella-Branger D, Cavenee WK, Ohgaki H, Wiestler OD, Kleihues P, Ellison DW (2016). The 2016 World Health Organization classification of tumors of the central nervous system: A summary. Acta Neuropathologica.

[17] Puchalski, R. B., Shah, N., Miller, J., Dalley, R., Nomura, S. R., Yoon, J. G., Smith, K. A., Lankerovich, M., Bertagnolli, D., Bickley, K., Boe, A. F., Brouner, K., Butler, S., Caldejon, S., Chapin, M., Datta, S., Dee, N., Desta, T., Dolbeare, T., Dotson, N., … Foltz, G. D. (2018). An anatomic transcriptional atlas of human glioblastoma. Science (New York, N.Y.), 360(6389), 660–663.10.1126/science.aaf2666 10.1126/science.aaf2666 PMC6414061 29748285

[18] Yekula A, Yekula A, Muralidharan K, Kang K, Carter BS, Balaj L (2020). Extracellular vesicles in glioblastoma tumor microenvironment. Frontiers in Immunology.

[19] Behnan, J., Finocchiaro, G., & Hanna, G. (2019, April 1). The landscape of the mesenchymal signature in brain tumours. *Brain*. Oxford University Press. 10.1093/brain/awz044 10.1093/brain/awz044 PMC6485274 30946477

[20] Perrin SL, Samuel MS, Koszyca B, Brown MP, Ebert LM, Oksdath M, Gomez GA (2019). Glioblastoma heterogeneity and the tumour microenvironment: Implications for preclinical research and development of new treatments. Biochemical Society transactions.

[21] Justus CR, Sanderlin EJ, Yang LV (2015). Molecular connections between cancer cell metabolism and the tumor microenvironment. International Journal of Molecular Sciences.

[22] Boedtkjer E, Pedersen SF (2020). The acidic tumor microenvironment as a driver of cancer. Annual Review of Physiology.

[23] Huang HW, Zuo C, Chen X, Peng YP, Qiu YH (2016). Effect of tyrosine hydroxylase overexpression in lymphocytes on the differentiation and function of T helper cells. International Journal of Molecular Medicine.

[24] An Z, Knobbe-Thomsen CB, Wan X, Fan QW, Reifenberger G, Weiss WA (2018). EGFR cooperates with EGFRvIII to recruit macrophages in glioblastoma. Cancer Research.

[25] Carnero, A., & Lleonart, M. (2016). The hypoxic microenvironment : A determinant of cancer stem cell evolution. *Inside The Cell*, 96–105. 10.1002/icl3.1039 10.1002/bies.201670911 27417124

[26] Pearson JRD, Regad T (2017). Targeting cellular pathways in glioblastoma multiforme. Signal Transduction and Targeted Therapy.

[27] Struve, N., Binder, Z. A., Stead, L. F., Brend, T., Bagley, S. J., Faulkner, C., Ott, L., Müller-Goebel, J., Weik, A-S., Hoffer, K., Krug, L., Rieckmann, T., Bubmann, L., Henze, M., Morrissette, J. J. D., Kurian, K. M., Schüller, U., Petersen, C., Rothkamm, K., … Kriegs, M. (2020). EGFRvIII upregulates DNA mismatch repair resulting in increased temozolomide sensitivity of MGMT promoter methylated glioblastoma. *Oncogene*, 3041–3055. 10.1038/s41388-020-1208-5 10.1038/s41388-020-1208-5 PMC7142016 32066879

[28] Berghoff AS, Kiesel B, Widhalm G, Wilhelm D, Rajky O, Kurscheid S, Kresl P, Wöhrer A, Marosi C, Hegi ME, Preusser M (2017). Correlation of immune phenotype with IDH mutation in diffuse glioma. Neuro-oncology.

[29] Richardson, L. G., Choi, B. D., & Curry, W. T. (2019). (R)-2-hydroxyglutarate drives immune quiescence in the tumor microenvironment of IDH-mutant gliomas. Translational cancer research, 8(Suppl 2), S167–S170. 10.21037/tcr.2019.01.08 10.21037/tcr.2019.01.08 PMC6448779 30956952

[30] Nandakumar, P., Mansouri, A., & Das, S. (2017, September 29). The role of ATRX in glioma biology. *Frontiers in Oncology*. 10.3389/fonc.2017.00236 10.3389/fonc.2017.00236 PMC5626857 29034211

[31] Bowie, M., Hariharan, S., Hostettler, J., Roso, K., He, Y., Pirozzi, C., Roskoski, M., Keir, S., Brown, M., Zhang, G., Gromeier, M., Yan, H., & Ashley, D. (2019). IMMU-34. ATRX mutations predict response to innate based therapy in glioma. *Neuro-Oncology*, 21(Suppl 6), vi126. 10.1093/neuonc/noz175.526

[32] Behling, F., & Schittenhelm, J. (2019, June 1). Oncogenic BRAF alterations and their role in brain tumors. *Cancers, 11*(6), 794. 10.3390/cancers11060794 10.3390/cancers11060794 PMC6627484 31181803

[33] Chen R, Keoni C, Waker CA, Lober RM, Gutmann DH (2019). KIAA1549-BRAF expression establishes a permissive tumor microenvironment through NFκB-mediated CCL2 production. Neoplasia (United States).

[34] Wang, Q., Hu, B., Hu, X., Kim, H., Squatrito, M., Scarpace, L., deCarvalho, A. C., Lyu, S., Li, P., Li, Y., Barthel, F., Cho, H. J., Lin, Y-H., Satani, N., Martinex-Ledesma, E., Zheng, S., Cheng, E., Sauve, C-E. G., Olar, … & Verhaak, R. G. W. (2017). Tumor evolution of glioma-intrinsic gene expression subtypes associates with immunological changes in the microenvironment. *Cancer Cell*, *32*(1), 42-56.e6. 10.1016/j.ccell.2017.06.003 10.1016/j.ccell.2017.06.003 PMC5599156 28697342

[35] Piro, G., Carbone, C., Carbognin, L., Pilotto, S., Ciccarese, C., Iacovelli, R., Milella, M., Bria E., & Tortora, G. (2019, October 1). Revising PTEN in the era of immunotherapy: New perspectives for an old story. *Cancers*, *11*(10), 1525. 10.3390/cancers11101525 10.3390/cancers11101525 PMC6826982 31658667

[36] Parsa AT, Waldron JS, Panner A, Crane CA, Parney IF, Barry JJ, Cachola KE, Murray JC, Tihan T, Jensen MC, Mischel PS, Stokoe D, Pieper RO (2007). Loss of tumor suppressor PTEN function increases B7–H1 expression and immunoresistance in glioma. Nature Medicine.

[37] Waldron JS, Yang I, Han S, Tihan T, Sughrue ME, Mills SA, Pieper RO, Parsa AT (2010). Implications for immunotherapy of tumor-mediated T-cell apoptosis associated with loss of the tumor suppressor PTEN in glioblastoma. Journal of clinical neuroscience : Official journal of the Neurosurgical Society of Australasia.

[38] Chen P, Zhao D, Li J, Liang X, Li J, Chang A, Henry VK, Lan Z, Spring DJ, Rao G, Wang YA, DePinho RA (2019). Symbiotic macrophage-glioma cell interactions reveal synthetic lethality in PTEN-Null glioma. Cancer Cell.

[39] Smith-Mungo LI, Kagan HM (1998). Lysyl oxidase: Properties, regulation and multiple functions in biology. Matrix Biology.

[40] Pore N, Liu S, Haas-Kogan DA, O’Rourke DM, Maity A (2003). PTEN mutation and epidermal growth factor receptor activation regulate vascular endothelial growth factor (VEGF) mRNA expression in human glioblastoma cells by transactivating the proximal VEGF promoter. Cancer Research.

[41] Oldrini, B., Vaquero-Siguero, N., Mu, Q., Kroon, P., Zhang, Y., Galán-Ganga, M., Bao, Z., Wang, Z., Liu, H., Sa, J. K., Zhao, J., Kim, H., Rodriguez-Perales, S., Nam, D-H., Verhaak, R. G. W., Rabadan, R., Jiang, T., Wang, J., & Squatrito, M. (n.d.). MGMT genomic rearrangements contribute to chemotherapy resistance in gliomas. *Nature Communications, 11,* 3883. 10.1038/s41467-020-17717-0 10.1038/s41467-020-17717-0 PMC7403430 32753598

[42] Pistollato F, Abbadi S, Rampazzo E, Persano L, Della Puppa A, Frasson C, Sarto E, Scienza R, D’avella D, Basso G (2010). Intratumoral hypoxic gradient drives stem cells distribution and MGMT expression in glioblastoma. Stem Cells.

[43] Wickström, M., Dyberg, C., Milosevic, J., Einvik, C., Calero, R., Sveinbjörnsson, B., Sandén, E., Darabi, A., Siesjö, P., Kool, M., Kogner, P., Baryawno, N., & Johnsen, J. I. (2015). Wnt/β-catenin pathway regulates MGMT gene expression in cancer and inhibition of Wnt signalling prevents chemoresistance. *Nature Communications*, *6*. 10.1038/ncomms9904 10.1038/ncomms9904 PMC4674781 26603103

[44] Zhao L, Zhang J, Xuan S, Liu Z, Wang Y, Zhao P (2021). Molecular and clinicopathological characterization of a prognostic immune gene signature associated with MGMT methylation in glioblastoma. Frontiers in Cell and Developmental Biology.

[45] Coppé, J. P., Patil, C. K., Rodier, F., Sun, Y., Muñoz, D. P., Goldstein, J., Nelson, P. S., Desprez, P-Y., & Campisi, J. (2008). Senescence-associated secretory phenotypes reveal cell-nonautonomous functions of oncogenic RAS and the p53 tumor suppressor. *PLoS Biology*, *6*(12). 10.1371/journal.pbio.0060301 10.1371/journal.pbio.0060301 PMC2592359 19053174

[46] Kastenhuber, E. R., & Lowe, S. W. (2017, September 7). Putting p53 in Context. *Cell*, *170*(6), 1062–1078. 10.1016/j.cell.2017.08.028 10.1016/j.cell.2017.08.028 PMC5743327 28886379

[47] Cooks T, Harris CC, Oren M (2014). Caught in the cross fire: P53 in inflammation. Carcinogenesis.

[48] Guo G, Yu M, Xiao W, Celis E, Cui Y (2017). Local activation of p53 in the tumor microenvironment overcomes immune suppression and enhances antitumor immunity. Cancer Research.

[49] Schröder, L. B. W., & McDonald, K. L. (2015). CDK4/6 inhibitor PD0332991 in glioblastoma treatment: Does it have a future? *Frontiers in Oncology*, *5*(NOV), 259. 10.3389/fonc.2015.00259 10.3389/fonc.2015.00259 PMC4663246 26649278

[50] Goldhoff P, Clarke J, Smirnov I, Berger MS, Prados MD, James CD, Perry A, Phillips JJ (2012). Clinical stratification of glioblastoma based on alterations in retinoblastoma tumor suppressor protein (RB1) and association with the proneural subtype. Journal of neuropathology and experimental neurology.

[51] Ciznadija D, Liu Y, Pyonteck SM, Holland EC, Koff A (2011). Cyclin D1 and Cdk4 mediate development of neurologically destructive oligodendroglioma. Cancer Research.

[52] Hambardzumyan D, Bergers G (2015). Glioblastoma: Defining tumor niches. TRENDS in CANCER.

[53] Griguer CE, Oliva CR, Gobin E, Marcorelles P, Benos DJ, Jack R, Gillespie GY (2008). CD133 is a marker of bioenergetic stress in human glioma. PLoS ONE.

[54] Goodman R, Slater E, Herschman HR (1980). Epidermal growth factor induces tyrosine hydroxylase in a clonal pheochromocytoma cell line, PC-G2. Journal of Cell Biology.

[55] Shergalis A, Bankhead A, Luesakul U, Muangsin N, Neamati N (2018). Current challenges and opportunities in treating glioblastomas. Pharmacological Reviews.

[56] Tsuchihashi, K., Okazaki, S., Ohmura, M., Ishikawa, M., Sampetrean, O., Onishi, N., Wakimoto. H., Yoshikawa, M., Seishima, R., Iwasaki, Y., Morikawa, T., Abe, S., Takao, A., Shimizu, M., Masuko, T., Nagane, M., Furnari, F. B., Akiyama. T., Suematsu., M., …& Nagano, O. (2016). The EGF receptor promotes the malignant potential of glioma by regulating amino acid transport system xc(−) HHS Public Access. *Cancer Res*, *76*(10), 2954–2963. 10.1158/0008-5472.CAN-15-2121 10.1158/0008-5472.CAN-15-2121 PMC4873328 26980765

[57] Conrad M, Sato H (2012). The oxidative stress-inducible cystine/glutamate antiporter, system x c-: Cystine supplier and beyond. Amino Acids.

[58] Corsi L, Mescola A, Alessandrini A (2019). Glutamate receptors and glioblastoma multiforme: An old “route” for new perspectives. International journal of molecular sciences.

[59] Al-Nedawi K, Meehan B, Micallef J, Lhotak V, May L, Guha A, Rak J (2008). Intercellular transfer of the oncogenic receptor EGFRvIII by microvesicles derived from tumour cells. Nature Cell Biology.

[60] Diksin M, Smith SJ, Rahman R (2017). The molecular and phenotypic basis of the glioma invasive perivascular niche. International Journal of Molecular Sciences.

[61] Hao Q, Vadgama JV, Wang P (2020). CCL2/CCR2 signaling in cancer pathogenesis. Cell Communication and Signaling.

[62] Bonavia R, Inda MM, Vandenberg S, Cheng SY, Nagane M, Hadwiger P, Tan P, Sah DWY, Cavanee WK, Furnari FB (2012). EGFRvIII promotes glioma angiogenesis and growth through the NF-B, interleukin-8 pathway. Oncogene.

[63] Guequén, A., Zamorano, P., Córdova, F., Koning, T., Torres, A., Ehrenfeld, P., Boric, M. P., Salazar-Onfray, F., Gavard, J., Duran, W. N., Quezada, C., Sarmiento, J., & Sánchez, F. A. (2019). Interleukin-8 secreted by glioblastoma cells induces microvascular hyperpermeability through no signaling involving S-Nitrosylation of VE-cadherin and p120 in endothelial cells. *Frontiers in Physiology*, *0*(JUL), 988. 10.3389/FPHYS.2019.00988 10.3389/fphys.2019.00988 PMC6694439 31440166

[64] Zanca, C., Villa, G. R., Benitez, J. A., Thorne, A. H., Koga, T., D’Antonio, M., Ikegami, S., Ma, J., Boyer, A. D., Banisadr, A., Jameson, N. M., Parisian, A. D., Eliseeva, O. V., Barnabe, G. F., Liu, F., Wu, S., Yang, H., Wykosky, J., Frazer, K. A., … Furnari, F. B. (2017). Glioblastoma cellular cross-talk converges on NF-κB to attenuate EGFR inhibitor sensitivity. *Genes and Development*, *31*(12), 1212–1227. 10.1101/gad.300079.117 10.1101/gad.300079.117 PMC5558924 28724615

[65] Lamano JB, Lamano JB, Li YD, DiDomenico JD, Choy W, Veliceasa D, Oyon DE, Fakurnejad S, Ampie L, Kesavabholta K, Kaur R, Kaur G, Biyashev D, Unruh DJ, Horbinski CM, James D, Parsa AT, Bloch O (2019). Glioblastoma-derived IL6 induces immunosuppressive peripheral myeloid cell PD-L1 and promotes tumor growth. Clinical Cancer Research.

[66] Litak J, Mazurek M, Grochowski C, Kamieniak P, Roliński J (2019). PD-L1/PD-1 axis in glioblastoma multiforme. International Journal of Molecular Sciences.

[67] Rius-Pérez, S., Pérez, S., Martí-Andrés, P., Monsalve, M., & Sastre, J. (2020). Nuclear factor Kappa B signaling complexes in acute inflammation. *Antioxidants & Redox Signaling*, ars.2019.7975. 10.1089/ars.2019.7975 10.1089/ars.2019.7975 31856585

[68] Cohen AL, Holmen SL, Colman H (2013). IDH1 and IDH2 mutations in gliomas. Current Neurol Neurosci Rep.

[69] Michelson N, Rincon-Torroella J, Quiñones-Hinojosa A, Greenfield JP (2016). Exploring the role of inflammation in the malignant transformation of low-grade gliomas. Journal of neuroimmunology.

[70] Batsios G, Viswanath P, Subramani E, Najac C, Gillespie AM, Santos RD, Molloy AR, Pieper RO, Ronen SM (2019). PI3K/mTOR inhibition of IDH1 mutant glioma leads to reduced 2HG production that is associated with increased survival. Scientific Reports.

[71] Perus, L. J. M., Walsh, L. A., & Walsh, L. A. (2019). Microenvironmental heterogeneity in brain malignancies. *Frontiers in Immunology*, *10*(October). 10.3389/fimmu.2019.02294 10.3389/fimmu.2019.02294 PMC6779728 31632393

[72] Barthel FP, Wesseling P, Verhaak RGW (2018). Reconstructing the molecular life history of gliomas. Acta Neuropathologica.

[73] Kechagia JZ, Ivaska J, Roca-Cusachs P (2019). Integrins as biomechanical sensors of the microenvironment. Nature Reviews Molecular Cell Biology.

[74] Previtali SC, Quattrini A, Pardini CL, Nemni R, Feltri ML, Boncinelli E, Canal N, Wrabetz L (1999). Laminin receptor α6β4 integrin is highly expressed in ENU-induced glioma in rat. Glia.

[75] Stewart, R. L., Chen, M., Mulkearns-Hubert, E. E., Lathia, J., O’Connor, K. L., & Horbinski, C. (2019). Integrin α6β4 is downregulated in mutant IDH1 oligodendrogliomas, promotes glioma growth, and associates with a worse outcome in glioma patients. *bioRxiv* [preprint]. 10.1101/726489 .

[76] Killela, P. J., Reitman, Z. J., Jiao, Y., Bettegowda, C., Agrawal, N., Diaz, L. A., Friedman, A. H., Friedman, H., Gallia, G. L., Giovanella, B. C., Grollman, A. P., He, T-C., He, Y., Hruban, R. H., Jallo, G. I., Mandahl, N., Meeker, A. K., Mertens, F., Netto, G. J., … &Yan, H. (2013). TERT promoter mutations occur frequently in gliomas and a subset of tumors derived from cells with low rates of self-renewal. *Proceedings of the National Academy of Sciences of the United States of America*, *110*(15), 6021–6026. 10.1073/pnas.1303607110 10.1073/pnas.1303607110 PMC3625331 23530248

[77] Korshunov A, Meyer J, Capper D, Christians A, Remke M, Witt H, Pfister S, von Deimling A, Hartmann C (2009). Combined molecular analysis of BRAF and IDH1 distinguishes pilocytic astrocytoma from diffuse astrocytoma. Acta neuropathologica.

[78] Knobbe CB, Reifenberger J, Reifenberger G (2004). Mutation analysis of the Ras pathway genes NRAS, HRAS, KRAS and BRAF in glioblastomas. Acta neuropathologica.

[79] Schindler G, Capper D, Meyer J, Janzarik W, Omran H, Herold-Mende C, Schmieder K, Wesseling P, Mawrin C, Hasselblatt M, Louis DN, Korshunov A, Pfister S, Hartmann C, Paulus W, Reifenberger G, Von Deimling A (2011). Analysis of BRAF V600E mutation in 1,320 nervous system tumors reveals high mutation frequencies in pleomorphic xanthoastrocytoma, ganglioglioma and extra-cerebellar pilocytic astrocytoma. Acta Neuropathologica.

[80] D’Angelo, F., Ceccarelli, M., Tala, Garofano, L., Zhang, J., Frattini, V., Caruso, F. P., Lewis, G., Alfar, K. D., Bauschet, L., Berzero, G., Cachia, D., Cangiano, M., Capelle, L., de Groot, J., DiMeco, F., Ducray, F., Farah, W., Finocchiaro, G., …& Iavarone, A. (2019). The molecular landscape of glioma in patients with neurofibromatosis 1. *Nature Medicine*, *25*(1), 176–187. 10.1038/s41591-018-0263-8 10.1038/s41591-018-0263-8 PMC6857804 30531922

[81] Lobbous M, Bernstock JD, Coffee E, Friedman GK, Metrock LK, Chagoya G, Elsayed G, Nakano I, Hackney JR, Korf BR, Nabors LB (2020). An update on neurofibromatosis type 1-associated gliomas. Cancers.

[82] Pulido, R., Baker, S. J., Barata, J. T., Carracedo, A., Cid, V. J., Chin-Sang, I. D., Dave, V., Hertog, J. D., Devreotes, P., Eickholt, B. J., Eng, C., Furnari, F. B., Georgesco, M-M., Gericke, A., Hopkins, B., Jiang, X., Lee, S-R., Losche, M., Malaney, P., … & Leslie, N. R. (2015). A unified nomenclature and amino acid numbering for human PTEN. *Sci Signal*, *7*(332), 15. 10.1126/scisignal.2005560 10.1126/scisignal.2005560 PMC4367864 24985344

[83] Brito C, Azevedo A, Esteves S, Marques AR, Martins C, Costa I, Mafra M, Marques JMN, Roque L, Pojo M (2019). Clinical insights gained by refining the 2016 WHO classification of diffuse gliomas with: EGFR amplification, TERT mutations, PTEN deletion and MGMT methylation. BMC Cancer.

[84] Cetintas, V. B., & Batada, N. N. (2020, January 30). Is there a causal link between PTEN deficient tumors and immunosuppressive tumor microenvironment? *Journal of Translational Medicine, 18,* 45. 10.1186/s12967-020-02219-w 10.1186/s12967-020-02219-w PMC6993356 32000794

[85] Peng, W., Qing Chen, J., Liu, C., Malu, S., Creasy, C., Tetzlaff, M. T., Xu, C., McKenzie, J. A., Zhang, C., Liang, X., Williams, L. J., Deng, W., Chen, G., Mbofung, R., Lazar, A. J., Torres-Cabala, C. A., Cooper, Z. A. Chen, P-L., Tieu, … Roszik, J. (2016). Loss of PTEN promotes resistance to T cell-mediated immunotherapy Analysis and interpretation of data (statistical analysis and bioinformatic analysis): HHS Public Access. *Cancer Discov*, *6*(2), 202–216. 10.1158/2159-8290.CD-15-0283 10.1158/2159-8290.CD-15-0283 PMC4744499 26645196

[86] Shao H, Chung J, Lee K, Balaj L, Min C, Carter BS, Hochberg FH, Breakefield XO, Lee H, Weissleder R (2015). Chip-based analysis of exosomal mRNA mediating drug resistance in glioblastoma. Nature Communications.

[87] Hegi ME, Diserens AC, Gorlia T, Hamou MF, De Tribolet N, Weller M, Kros JM, Hainfellner JA, Mason W, Mariani L, Bromberg JEC, Hau P, Mirimanoff RO, Cairncross JG, Janzer RC, Stupp R (2005). MGMT gene silencing and benefit from temozolomide in glioblastoma. New England Journal of Medicine.

[88] Ng, L., Kaur, P., Bunnag, N., Suresh, J., Sung, I., Tan, Q., Gruber, J., & Tolwinski, N. (2019). WNT Signaling in Disease. *Cells*, *8*(8), 826. 10.3390/cells8080826 10.3390/cells8080826 PMC6721652 31382613

[89] Lujambio A, Akkari L, Simon J, Grace D, Tschaharganeh DF, Bolden JE, Zhao Z, Thapar V, Joyce JA, Krizhanovsky V, Lowe SW (2013). Non-cell-autonomous tumor suppression by p53. Cell.

[90] Xue W, Zender L, Miething C, Dickins RA, Hernando E, Krizhanovsky V, Cordon-Cardo C, Lowe SW (2007). Senescence and tumour clearance is triggered by p53 restoration in murine liver carcinomas. Nature.

[91] Ham SW, Jeon HY, Jin X, Kim EJ, Kim JK, Shin YJ, Lee Y, Kim SH, Lee SY, Seo S, Park MG, Kim H-M, Nam D-H, Kim H (2019). TP53 gain-of-function mutation promotes inflammation in glioblastoma. Cell Death and Differentiation.

[92] Zhang, Y., Dube, C., Gibert, M., Cruickshanks, N., Wang, B., Coughlan, M., Yang, Y., Setiady, I., Deveau, C., Saoud, K., Grello, C., Oxford, M., Yuan, F., & Abounader, R. (2018, September 1). The p53 pathway in glioblastoma. *Cancers, 10*(9), 297. 10.3390/cancers10090297 10.3390/cancers10090297 PMC6162501 30200436

[93] Saleh T, Tyutynuk-Massey L, Cudjoe EK, Idowu MO, Landry JW, Gewirtz DA (2018). Non-cell autonomous effects of the senescence-associated secretory phenotype in cancer therapy. Frontiers in oncology.

[94] Fujita, K. (2019). P53 isoforms in cellular senescence-and ageing-associated biological and physiological functions. *International Journal of Molecular Sciences*, *20*(23). 10.3390/ijms20236023 10.3390/ijms20236023 PMC6928910 31795382

[95] Biasoli D, Sobrinho MF, Da Fonseca ACC, De Matos DG, Romão L, De Moraes Maciel R, Rehen SK, Moura-Neto V, Borges HL, Lima FRS (2014). Glioblastoma cells inhibit astrocytic p53-expression favoring cancer malignancy. Oncogenesis.

[96] Akhavan A, Griffith OL, Soroceanu L, Leonoudakis D, Luciani-Torres MG, Daemen A, Gray JW, Muschler JL (2012). Loss of cell-surface laminin anchoring promotes tumor growth and is associated with poor clinical outcomes. Cancer Research.

[97] Ohnishi, T., Arita, N., Hiraga, S., Higuchi, M., & Hayakawa, T. (1991). Human malignant glioma cells migrate to fibronectin and laminin: Role of extracellular matrix components in glioma cell invasion. *Biological Aspects of Brain Tumors*, Vol 1 (408–415. 10.1007/978-4-431-68150-2_57

[98] Murphree AL, Benedict WF (1984). Retinoblastoma: Clues to human oncogenesis. Science.

[99] Bronner, S. M., Merrick, K. A., Murray, J., Salphati, L., Moffat, J. G., Pang, J., Sneeringer, C. J., Dompe, N., Cyr, P., Purkey, H., de Leon Boenig, G., Li, J., Kolesnikov, A., Larouche-Gauthier, R., Lai, K. W., Shen, X, Aubert-Nicol, S., Chen, Y-C., … Heffron, T. P. (2019). Design of a brain-penetrant CDK4/6 inhibitor for glioblastoma. *Bioorganic and Medicinal Chemistry Letters*, *29*(16), 2294–2301. 10.1016/j.bmcl.2019.06.021 10.1016/j.bmcl.2019.06.021 31307887

[100] Henson JW, Schnitker BL, Correa KM, von Deimling A, Fassbender F, Xu H-J, Benedict WF, Yandell DW, Louis DN (1994). The retinoblastoma gene is involved in malignant progression of astrocytomas. Annals of Neurology.

[101] Kreis NN, Louwen F, Yuan J (2019). The multifaceted p21 (Cip1/Waf1/CDKN1A) in cell differentiation, migration and cancer therapy. Cancers.

[102] Warfel NA, El-Deiry WS (2013). P21WAF1 and tumourigenesis: 20 years after. Current Opinion in Oncology.

[103] Zhang D, Dai D, Zhou M, Li Z, Wang C, Lu Y, Wang J (2018). Inhibition of cyclin D1 expression in human glioblastoma cells is associated with increased temozolomide chemosensitivity. Cellular Physiology and Biochemistry.

[104] Schneider SW, Ludwig T, Tatenhorst L, Braune S, Oberleithner H, Senner V, Paulus W (2004). Glioblastoma cells release factors that disrupt blood-brain barrier features. Acta neuropathologica.

[105] Pinton L, Masetto E, Vettore M, Solito S, Magri S, D’Andolfi M, Bianco PD, Lollo G, Benoit J-P, Okada H, Diaz A, Puppa AD, Mandruzzato S (2019). The immune suppressive microenvironment of human gliomas depends on the accumulation of bone marrow-derived macrophages in the center of the lesion. Journal for ImmunoTherapy of Cancer.

[106] Dix AR, Brooks WH, Roszman TL, Morford LA (1999). Immune defects observed in patients with primary malignant brain tumors. Journal of Neuroimmunology.

[107] Chen X, Fan X, Zhao C, Zhao Z, Hu L, Wang D, Wang R, Fang Z (2020). Molecular subtyping of glioblastoma based on immune-related genes for prognosis. Scientific Reports.

[108] Huang S, Song Z, Zhang T, He X, Huang K, Zhang Q, Shen J, Pan J (2020). Identification of immune cell infiltration and immune-related genes in the tumor microenvironment of glioblastomas. Frontiers in Immunology.

[109] Mitchell DA, Xie W, Schmittling R, Learn C, Friedman A, McLendon RE, Sampson JH (2008). Sensitive detection of human cytomegalovirus in tumors and peripheral blood of patients diagnosed with glioblastoma. Neuro-Oncology.

[110] Quail DF, Joyce JA (2017). The microenvironmental landscape of brain tumors. Cancer Cell.

[111] Chen H, Li M, Guo Y, Zhong Y, He Z, Xu Y, Zou J (2020). Immune response in glioma’s microenvironment. Innovative Surgical Sciences.

[112] Xu, J., Zhang, J., Zhang, Z., Gao, Z., Qi, Y., Qiu, W., Pan, Z., Guo, Q., Li, B., Zhao, S., Guo, X., Qian, M., Chen, Z., Wang, S., Gao, X., Zhang, S., Wang, H., Guo, Z., Zhang, P., …& Li, G. (2021). Hypoxic glioma-derived exosomes promote M2-like macrophage polarization by enhancing autophagy induction. *Cell Death & Disease*, *12*(4), 1–16. 10.1038/s41419-021-03664-1 10.1038/s41419-021-03664-1 PMC8026615 33828078

[113] Wang, Q., Hu, X., Muller, F., Kim, H., Squatrito, M., Mikkelsen, T., Scarpace, L., Barthel, F., Lin, Y-H., Satani, S., Martinez-Ledesma, E., Chang, E., Olar, A., Hu, B., deCarvalho, A., Eskilsson, E., Zheng, S., Heimberger, A., Sulman, E., … & Verhaak, R. (2017). Tumor evolution of glioma intrinsic gene expression subtype associates with immunological changes in the microenvironment. *Cancer Cell*, *18*(suppl_6), vi202–vi202. 10.1093/neuonc/now212.854

[114] Hao Z, Guo D (2019). EGFR mutation: Novel prognostic factor associated with immune infiltration in lower-grade glioma; an exploratory study. BMC Cancer.

[115] Li F, Zhang W, Wang M, Jia P (2020). IL1RAP regulated by PRPRD promotes gliomas progression via inducing neuronal synapse development and neuron differentiation in vitro. Pathology Research and Practice.

[116] Scherer HJ (1938). Structural development in gliomas. American Journal of Cancer.

[117] Singh SK, Clarke ID, Terasaki M, Bonn VE, Hawkins C, Squire J, Dirks PB (2003). Identification of a cancer stem cell in human brain tumors. CANCER RESEARCH.

[118] Bao S, Wu Q, Sathornsumetee S, Hao Y, Li Z, Hjelmeland AB, Shi Q, McLendon RE, Bigner DD, Rich JN (2006). Stem cell – like glioma cells promote tumor angiogenesis through vascular endothelial growth factor. Cancer Research.

[119] Calabrese C, Poppleton H, Kocak M, Hogg TL, Fuller C, Hamner B, Oh EY, Gaber MW, Finklestein D, Allen M, Frank A, Bayazitove IT, Zakharenko SS, Gajjar A, Davidoff A, Gilbertson RJ (2007). A perivascular niche for brain tumor stem cells. Cancer Cell.

[120] Wen L, Tan Y, Dai S, Zhu Y, Meng T, Yang X, Liu X, Yuan H, Hu F (2017). Vegf-mediated tight junctions pathological fenestration enhances doxorubicin-loaded glycolipid-like nanoparticles traversing bbb for glioblastoma-targeting therapy. Drug Delivery.

[121] Gruys, E., Toussaint, M. J. M., Niewold, T. A., & Koopmans, S. J. (2005). Acute phase reaction and acute phase proteins. *Journal of Zhejiang University: Science*, *6 B*(11), 1045–1056. 10.1631/jzus.2005.B1045 10.1631/jzus.2005.B1045 PMC1390650 16252337

[122] West, A. J., Tsui, V., Stylli, S. S., Nguyen, H. P. T., Morokoff, A. P., Kaye, A. H., & Luwor, R. B. (2018, October 1). The role of interleukin-6-STAT3 signalling in glioblastoma. *Oncology Letters*. Spandidos Publications. 10.3892/ol.2018.9227 10.3892/ol.2018.9227 PMC6144698 30250528

[123] Hasan T, Caragher SP, Shireman JM, Park CH, Atashi F, Baisiwala S, Lee G, Guo D, Wang JY, Dey M, Wu M, Lesniak MS, Horbinski CM, James D, Ahmed AU (2019). Interleukin-8/CXCR2 signaling regulates therapy-induced plasticity and enhances tumorigenicity in glioblastoma. Cell Death and Disease.

[124] Raychaudhuri B, Vogelbaum MA (2011). IL-8 is a mediator of NF-κB induced invasion by gliomas. Journal of Neuro-Oncology.

[125] Jögi A, Øra I, Nilsson H, Lindeheim Å, Makino Y, Poellinger L, Alexson H, Påhlman S (2002). Hypoxia alters gene expression in human neuroblastoma cells toward an immature and neural crest-like phenotype. Proceedings of the National Academy of Sciences of the United States of America.

[126] Kim KB, Choi YH, Kim IK, Chung CW, Kim BJ, Park YM, Jung YK (2002). Potentiation of Fas- and trail-mediated apoptosis by IFN-γ in A549 lung epithelial cells: Enhancement of caspase-8 expression through IFN-response element. Cytokine.

[127] Kominsky S, Johnson HM, Bryan G, Tanabe T, Hobeika AC, Subramaniam PS, Torres B (1998). IFNγ inhibition of cell growth in glioblastomas correlates with increased levels of the cyclin dependent kinase inhibitor p21(WAF1/CIP1). Oncogene.

[128] Corbet C, Feron O (2017). Tumour acidosis: From the passenger to the driver’s seat. Nature Reviews Cancer.

[129] Watkins S, Robel S, Kimbrough IF, Robert SM, Ellis-Davies G, Sontheimer H (2014). Disruption of astrocyte-vascular coupling and the blood-brain barrier by invading glioma cells. Nature Communications.

[130] Cheng L, Huang Z, Zhou W, Wu Q, Donnola S, Liu JK, Fang X, Sloan AE, Mao Y, Lathia JD, Min W, McLendon RE, Rich JN, Bao S (2013). Glioblastoma stem cells generate vascular pericytes to support vessel function and tumor growth. Cell.

[131] Saba R, Alsayed A, Zacny JP, Dudek AZ (2016). The Role of Forkhead Box Protein M1 in breast cancer progression and resistance to therapy. International journal of breast cancer.

[132] Wang S, Chen C, Li J, Xu X, Chen W, Li F (2020). The CXCL12 / CXCR4 axis confers temozolomide resistance to human glioblastoma cells via up-regulation of FOXM1. Journal of the Neurological Sciences.

[133] Thakur, B. K., Zhang, H., Becker, A., Matei, I., Huang, Y., Costa-Silva, B., Zheng, Y., Hoshino, A., Brazier, H., Xiang, J., Williams, C., Rodriguez-Barrueco, R., Silva, J. M., Zhang, W., Hearn, S., Elemento, O., Paknejad, N., Manova-Todorova, K., Welte, K., … Jacqueline Bromberg Lyden, D. (2014, April 8). Double-stranded DNA in exosomes: A novel biomarker in cancer detection. *Cell Research, 24,* 766–769. 10.1038/cr.2014.44 10.1038/cr.2014.44 PMC4042169 24710597

[134] Simon T, Jackson E, Giamas G (2020). Breaking through the glioblastoma micro-environment via extracellular vesicles. Oncogene.

[135] Bronisz A, Wang Y, Nowicki MO, Peruzzi P, Ansari KIA, Ogawa D, Balaj L, De Rienzo G, Mineo M, Nakano I, Ostrovski MC, Hochberg F, Weissleder R, Lawler SE, Chiocca EA, Godlewski J (2014). Extracellular vesicles modulate the glioblastoma microenvironment via a tumor suppression signaling network directed by miR-1. Cancer research.

[136] Hallal S, Mallawaaratchy DM, Wei H, Ebrahimkhani S, Stringer BW, Day BW, Boyd AW, Guillemin GJ, Buckland ME, Kaufman KL (2019). Extracellular vesicles released by glioblastoma cells stimulate normal astrocytes to acquire a tumor-supportive phenotype via p53 and MYC signaling pathways. Molecular Neurobiology.

[137] Choi D, Montermini L, Kim DK, Meehan B, Roth FP, Rak J (2018). The impact of oncogenic egfrviii on the proteome of extracellular vesicles released from glioblastoma cells. Molecular and Cellular Proteomics.

[138] Skog J, Wurdinger T, van Rijn S, Meijer D, Gainche L, Curry WT, Carter BS, Krichevsky AM, Breakefield XO (2008). Glioblastoma microvesicles transport RNA and protein that promote tumor growth and provide diagnostic biomarkers. Nature Cell Bbiology.

[139] Figueroa, J. M., Skog, J., Akers, J., Li, H., Komotar, R., Jensen, R., Ringel, F., Yang, I., Kalkanis, S., Thompson, R., LoGuidice, L., Berghoff, E., Parsa, A., Liau, L., Curry, W., Cahill, D., Bettegowda, C., Lang, F. F., Chiocca, E. A., … Carter, B. S. (2017). Detection of wild-type EGFR amplification and EGFRvIII mutation in CSF-derived extracellular vesicles of glioblastoma patients. *Neuro-oncology*, *19*(11), 1494–1502. 10.1093/NEUONC/NOX085 10.1093/neuonc/nox085 PMC5737576 28453784

[140] Taraboletti G, D’Ascenzo S, Giusti I, Marchetti D, Borsotti P, Millimaggi D, Giavazzi R, Pavan A, Dolo V (2006). Bioavailability of VEGF in tumor-shed vesicles depends on vesicle burst induced by acidic pH 1. Neoplasia.

[141] McGranahan T, Therkelsen KE, Ahmad S, Nagpal S (2019). Current state of immunotherapy for treatment of glioblastoma. Current treatment options in oncology.

[142] Huang B, Li X, Li Y, Zhang J, Zong Z, Zhang H (2021). Current immunotherapies for glioblastoma multiforme. Frontiers in immunology.

[143] Brown CE, Badie B, Barish ME, Weng L, Ostberg JR, Chang WC, Naranjo A, Starr R, Wagner J, Wright C, Zhai Y, Bading JR, Ressler JA, Portnow J, D’Apuzzo M, Forman SJ, Jensen MC (2015). Bioactivity and safety of IL13Rα2-redirected chimeric antigen receptor CD8+ T cells in patients with recurrent glioblastoma. Clinical cancer research : An official journal of the American Association for Cancer Research.

[144] Ahmed, N., Brawley, V., Hegde, M., Bielamowicz, K., Kalra, M., Landi, D., Robertson, C., Gray, T. L., Diouf, O., Wakefield, A., Ghazi, A., Gerken, C., Yi, Z., Ashoori, A., Wu, M. F., Liu, H., Rooney, C., Dotti, G., Gee, A., Su, J., … Gottschalk, S. (2017). HER2-specific chimeric antigen receptor-modified virus-specific T cells for progressive glioblastoma: A phase 1 dose-escalation trial. *JAMA Oncology*, 3(8), 1094–1101. 10.1001/jamaoncol.2017.0184 10.1001/jamaoncol.2017.0184 PMC5747970 28426845

[145] Schuessler A, Smith C, Beagley L, Boyle GM, Rehan S, Matthews K, Jones L, Crough T, Dasari V, Klein K, Smalley A, Alexander H, Walker DG, Khanna R (2014). Autologous T-cell therapy for cytomegalovirus as a consolidative treatment for recurrent glioblastoma. Cancer research.

[146] Kong, D. S., Nam, D. H., Kang, S. H., Lee, J. W., Chang, J. H., Kim, J. H., Lim, Y. J., Koh, Y. C., Chung, Y. G., Kim, J. M., & Kim, C. H. (2017). Phase III randomized trial of autologous cytokine-induced killer cell immunotherapy for newly diagnosed glioblastoma in Korea. *Oncotarget*, 8(4), 7003–7013. 10.18632/oncotarget.12273 10.18632/oncotarget.12273 PMC5351686 27690294

[147] Margolin K, Ernstoff MS, Hamid O, Lawrence D, McDermott D, Puzanov I, Wolchok JD, Clark JI, Sznol M, Logan TF, Richards J, Michener T, Balogh A, Heller KN, Hodi FS (2012). Ipilimumab in patients with melanoma and brain metastases: An open-label, phase 2 trial. The Lancet. Oncology.

[148] Reardon DA, Brandes AA, Omuro A, Mulholland P, Lim M, Wick A, Baehring J, Ahluwalia MS, Roth P, Bähr O, Phuphanich S, Sepulveda JM, De Souza P, Sahebjam S, Carleton M, Tatsuoka K, Taitt C, Zwirtes R, Sampson J, Weller M (2020). Effect of nivolumab vs bevacizumab in patients with recurrent glioblastoma: The CheckMate 143 phase 3 randomized clinical trial. JAMA Oncology.

[149] Rampling R, Peoples S, Mulholland PJ, James A, Al-Salihi O, Twelves CJ, McBain C, Jefferies S, Jackson A, Stewart W, Lindner J, Kutscher S, Hilf N, McGuigan L, Peters J, Hill K, Schoor O, Singh-Jasuja H, Halford SE, Ritchie JW (2016). A Cancer Research UK first time in human phase I trial of IMA950 (novel multipeptide therapeutic vaccine) in patients with newly diagnosed glioblastoma. Clinical Cancer Research: An Official Journal of the American Association for Cancer Research.

[150] Inogés S, Tejada S, de Cerio AL, Gállego Pérez-Larraya J, Espinós J, Idoate MA, Domínguez PD, de Eulate RG, Aristu J, Bendandi M, Pastor F, Alonso M, Andreu E, Cardoso FP, Valle RD (2017). A phase II trial of autologous dendritic cell vaccination and radiochemotherapy following fluorescence-guided surgery in newly diagnosed glioblastoma patients. Journal of translational medicine.

[151] Weller, M., Butowski, N., Tran, D. D., Recht, L. D., Lim, M., Hirte, H., Ashby, L., Mechtler, L., Goldlust, S. A., Iwamoto, F., Drappatz, J., O’Rourke, D. M., Wong, M., Hamilton, M. G., Finocchiaro, G., Perry, J., Wick, W., Green, J., He, Y., Turner, C. D., … ACT IV trial investigators (2017). Rindopepimut with temozolomide for patients with newly diagnosed, EGFRvIII-expressing glioblastoma (ACT IV): A randomised, double-blind, international phase 3 trial. *The Lancet. Oncology*, 18(10), 1373–1385. 10.1016/S1470-2045(17)30517-X 10.1016/S1470-2045(17)30517-X 28844499

[152] Schuster J, Lai RK, Recht LD, Reardon DA, Paleologos NA, Groves MD, Mrugala MM, Jensen R, Baehring JM, Sloan A, Archer GE, Bigner DD, Cruickshank S, Green JA, Keler T, Davis TA, Heimberger AB, Sampson JH (2015). A phase II, multicenter trial of rindopepimut (CDX-110) in newly diagnosed glioblastoma: The ACT III study. Neuro-oncology.

[153] Bloch O, Crane CA, Fuks Y, Kaur R, Aghi MK, Berger MS, Butowski NA, Chang SM, Clarke JL, McDermott MW, Prados MD, Sloan AE, Bruce JN, Parsa AT (2014). Heat-shock protein peptide complex-96 vaccination for recurrent glioblastoma: A phase II, single-arm trial. Neuro-oncology.

[154] Fenstermaker RA, Ciesielski MJ, Qiu J, Yang N, Frank CL, Lee KP, Mechtler LR, Belal A, Ahluwalia MS, Hutson AD (2016). Clinical study of a survivin long peptide vaccine (SurVaxM) in patients with recurrent malignant glioma. Cancer immunology, immunotherapy : CII.

[155] Batich KA, Reap EA, Archer GE, Sanchez-Perez L, Nair SK, Schmittling RJ, Norberg P, Xie W, Herndon JE, Healy P, McLendon RE, Friedman AH, Friedman HS, Bigner D, Vlahovic G, Mitchell DA, Sampson JH (2017). Long-term survival in glioblastoma with cytomegalovirus pp65-targeted vaccination. Clinical Cancer Research : An official journal of the American Association for Cancer Research.

[156] Curry WT, Gorrepati R, Piesche M, Sasada T, Agarwalla P, Jones PS, Gerstner ER, Golby AJ, Batchelor TT, Wen PY, Mihm MC, Dranoff G (2016). Vaccination with irradiated autologous tumor cells mixed with irradiated GM-K562 cells stimulates antitumor immunity and T lymphocyte activation in patients with recurrent malignant glioma. Clinical Cancer Research : An official journal of the American Association for Cancer Research.

[157] Liau, L. M., Ashkan, K., Tran, D. D., Campian, J. L., Trusheim, J. E., Cobbs, C. S., Heth, J. A., Salacz, M., Taylor, S., D’Andre, S. D., Iwamoto, F. M., Dropcho, E. J., Moshel, Y. A., Walter, K. A., Pillainayagam, C. P., Aiken, R., Chaudhary, R., Goldlust, S. A., Bota, D. A., Duic, P., … Bosch, M. L. (2018). First results on survival from a large Phase 3 clinical trial of an autologous dendritic cell vaccine in newly diagnosed glioblastoma*. Journal of translational medicine*, 16(1), 142. 10.1186/s12967-018-1507-6 10.1186/s12967-018-1507-6 PMC5975654 29843811

[158] Platten, M., Bunse, L., Wick, A., Bunse, T., Le Cornet, L., Harting, I., Sahm, F., Sanghvi, K., Tan, C. L., Poschke, I., Green, E., Justesen, S., Behrens, G. A., Breckwoldt, M. O., Freitag, A., Rother, L. M., Schmitt, A., Schnell, O., Hense, J., Misch, M., … Wick, W. (2021). A vaccine targeting mutant IDH1 in newly diagnosed glioma. Nature, 592(7854), 463–468. 10.1038/s41586-021-03363-z 10.1038/s41586-021-03363-z PMC8046668 33762734

[159] Lang, F. F., Conrad, C., Gomez-Manzano, C., Yung, W., Sawaya, R., Weinberg, J. S., Prabhu, S. S., Rao, G., Fuller, G. N., Aldape, K. D., Gumin, J., Vence, L. M., Wistuba, I., Rodriguez-Canales, J., Villalobos, P. A., Dirven, C., Tejada, S., Valle, R. D., Alonso, M. M., Ewald, B., … Fueyo, J. (2018). Phase I study of DNX-2401 (delta-24-RGD) oncolytic adenovirus: Replication and immunotherapeutic effects in recurrent malignant glioma. *Journal of clinical oncology*: official journal of the American Society of Clinical Oncology, 36(14), 1419–1427. 10.1200/JCO.2017.75.8219 10.1200/JCO.2017.75.8219 PMC6075856 29432077

[160] Weller, M., van den Bent, M., Preusser, M., Le Rhun, E., Tonn, J. C., Minniti, G., Bendszus, M., Balana, C., Chinot, O., Dirven, L., French, P., Hegi, M. E., Jakola, A. S., Platten, M., Roth, P., Rudà, R., Short, S., Smits, M., Taphoorn, M., von Deimling, A., … Wick, W. (2021). EANO guidelines on the diagnosis and treatment of diffuse gliomas of adulthood. *Nature reviews. Clinical oncology*, 18(3), 170–186. 10.1038/s41571-020-00447-z 10.1038/s41571-020-00447-z PMC7904519 33293629

